# The Bouma law accounts for crowding in 50 observers

**DOI:** 10.1167/jov.23.8.6

**Published:** 2023-08-04

**Authors:** Jan W. Kurzawski, Augustin Burchell, Darshan Thapa, Jonathan Winawer, Najib J. Majaj, Denis G. Pelli

**Affiliations:** 1 Department of Psychology, New York University, New York, NY, USA; 2 Cognitive Science & Computer Science, Swarthmore College, Swarthmore, PA, USA; 3 Center for Neural Science, New York University, New York, NY, USA; 4 Department of Psychology, New York University, New York, NY, USA; 5 Center for Neural Science, New York University, New York, NY, USA; 6 Center for Neural Science, New York University, New York, NY, USA; 7 Department of Psychology, New York University, New York, NY, USA; 8 Center for Neural Science, New York University, New York, NY, USA

**Keywords:** crowding, critical spacing, crowding distance, bouma's law, object recognition, statistics of crowding, asymmetries around the visual field

## Abstract

*Crowding* is the failure to recognize an object due to surrounding clutter. Our visual crowding survey measured 13 crowding distances (or “critical spacings”) twice in each of 50 observers. The survey includes three eccentricities (0, 5, and 10 deg), four cardinal meridians, two orientations (radial and tangential), and two fonts (Sloan and Pelli). The survey also tested foveal acuity, twice. Remarkably, fitting a two-parameter model—the well-known Bouma law, where crowding distance grows linearly with eccentricity—explains 82% of the variance for all 13 × 50 measured log crowding distances, cross-validated. An enhanced Bouma law, with factors for meridian, crowding orientation, target kind, and observer, explains 94% of the variance, again cross-validated. These additional factors reveal several asymmetries, consistent with previous reports, which can be expressed as crowding-distance ratios: 0.62 horizontal:vertical, 0.79 lower:upper, 0.78 right:left, 0.55 tangential:radial, and 0.78 Sloan-font:Pelli-font. Across our observers, peripheral crowding is independent of foveal crowding and acuity. Evaluation of the Bouma factor, *b* (the slope of the Bouma law), as a biomarker of visual health would be easier if there were a way to compare results across crowding studies that use different methods. We define a *standardized Bouma factor b*′ that corrects for differences from Bouma's 25 choice alternatives, 75% threshold criterion, and linearly symmetric flanker placement. For radial crowding on the right meridian, the standardized Bouma factor *b*′ is 0.24 for this study, 0.35 for Bouma (1970), and 0.30 for the geometric mean across five representative modern studies, including this one, showing good agreement across labs, including Bouma's. Simulations, confirmed by data, show that peeking can skew estimates of crowding (e.g., greatly decreasing the mean or doubling the *SD* of log *b*). Using gaze tracking to prevent peeking, individual differences are robust, as evidenced by the much larger 0.08 *SD* of log *b* across observers than the mere 0.03 test–retest SD of log *b* measured in half an hour. The ease of measurement of crowding enhances its promise as a biomarker for dyslexia and visual health.

## Introduction

Crowding is the failure to recognize an object due to surrounding clutter (Bouma, 1970; Bouma, 1973; Pelli, Palomares, & Majaj, 2004; Pelli & Tillman, 2008; Strasburger, Harvey, & Rentschler, 1991; Stuart & Burian, 1962). Crowding has been studied with several different tasks, including letter identification (Bouma, 1970; Flom, Heath, & Takahashi, 1963; Strasburger et al., 1991), Landolt rings (Flom, Heath, et al., 1963; Flom, Weymouth, & Kahneman, 1963), Vernier acuity (Levi, Klein, & Aitsebaomo, 1985; Malania, Herzog, & Westheimer, 2007; Westheimer & Hauske, 1975), face recognition (Farzin, Rivera, & Whitney, 2009; Louie, Bressler, & Whitney, 2007; Martelli, Majaj, & Pelli, 2005), and orientation discrimination (Andriessen & Bouma, 1976; Parkes, Lund, Angelucci, Solomon, & Morgan, 2001; Toet & Levi, 1992; Westheimer, Shimamura, & McKee, 1976). It is invariant with the size of target and flankers (Levi & Carney, 2009; Pelli et al., 2004; Pelli, Tillman, Freeman, Su, Berger, & Majaj, 2007; Strasburger et al., 1991; Tripathy & Cavanagh, 2002). Crowding is usually measured by sandwiching the target between two similar flanking objects, or *flankers*, and is characterized by the *crowding distance* (or “critical spacing”), which is the center-to-center distance from target to flanker at which recognition attains a criterion level of performance. Crowding distance increases linearly with eccentricity (Bouma, 1970; Kooi, Toet, Tripathy, & Levi, 1994; Levi & Carney, 2009; Pelli et al., 2004; Toet & Levi, 1992), and increases with target-flanker similarity (Andriessen & Bouma, 1976; Chastain, 1982; Kooi et al., 1994; Leat, Li, & Epp, 1999; Nazir, 1992; Pelli et al., 2004), as well as the number of distractors (Grainger, Tydgat, & Issele, 2010; Strasburger et al., 1991). Crowding also occurs for moving stimuli (Bex & Dakin, 2005; Bex, Dakin, & Simmers, 2003). For a review of the crowding literature, see Herzog, Sayim, Chicherov, and Manassi (2015), Levi (2008), Pelli and Tillman (2008), Strasburger (2020), Strasburger, Rentschler, and Juttner (2011), and Whitney and Levi (2011). Among the normally sighted, crowding was first reported in the periphery and, after some debate, has now been convincingly demonstrated in the fovea (Atkinson, Pimm-Smith, Evans, Harding, & Braddick, 1986; Coates, Levi, Touch, & Sabesan, 2018; Flom, Heath, et al., 1963; Liu & Arditi, 2000; Malania et al., 2007; Pelli et al., 2016; Siderov, Waugh, & Bedell, 2013; Toet & Levi, 1992).

We are interested in relating psychophysical measures of crowding to brain physiology, especially cortical magnification measured by functional magnetic resonance imaging (fMRI) in areas V1, V2, V3, and hV4. For this purpose, we tested crowding in 50 observers to characterize the statistics of crowding within and across individuals. The comparisons with fMRI are reported separately (Kurzawski, Pelli, & Winawer, 2021). Here, we report only the psychophysics. We tested with letters, which, after little or no training, provide the many possible targets that are needed for quick testing (Pelli & Robson, 1991). Long term, we are interested in testing crowding in children (Waugh, Pelli, Álvaro, & Formankiewicz, 2018) as an early biomarker for susceptibility to visual problems such as dyslexia. Crowding distance is highly conserved across object kind (Kooi et al., 1994; Pelli & Tillman, 2008), which suggests that letters, vernier, and Gabors might have similar crowding distances, but Grainger et al. (2010) reported different crowding distances for letters and symbols.

Crowding exhibits several striking asymmetries. Crowding distance measured radially (along a line passing through the foveal center) is roughly twice that measured tangentially, the orthogonal orientation (Greenwood, Szinte, Sayim, & Cavanagh, 2017; Kwon, Bao, Millin, & Tjan, 2014; Pelli, 2008; Petrov & Meleshkevich, 2011; Toet & Levi, 1992). Crowding distance has often been reported to be smaller in the lower than upper visual field (Fortenbaugh, Silver, & Robertson, 2015; Greenwood et al., 2017; He, Cavanagh, & Intriligator, 1996; Petrov & Meleshkevich, 2011) and on the horizontal than vertical midline (Chung, 2013; Coates, Ludowici, & Chung, 2021; Liu, Jiang, Sun, & He, 2009; Petrov & Meleshkevich, 2011; Toet & Levi, 1992; Wallis & Bex, 2012).

Crowding distance is a potentially valuable biomarker for several reasons. Crowding severely limits what we see and how fast we read, and it is associated with dyslexia. There are large individual differences in crowding distance and correspondingly large physiological differences in the sizes of relevant areas of visual cortex, which invite analysis by correlation (Kurzawski et al., 2021). Here, we measured crowding in 50 observers. Previous in-person crowding surveys (Grainger et al., 2010; Greenwood et al., 2017; Petrov & Meleshkevich, 2011; Toet & Levi, 1992) included at most 27 observers. The only remote crowding survey tested 793 observers but did not report any asymmetries (Li, Joo, Yeatman, & Reinecke, 2020). The above cited works used various kinds of stimuli, including letters of various fonts. The original reports of the crowding phenomenon were mostly letter based (Anstis, 1974; Bouma, 1970; Bouma, 1973; Ehlers, 1936; Ehlers, 1953; Korte, 1923; Stuart & Burian, 1962). Historical review of crowding is described elsewhere (Levi, 2008; Pelli et al., 2004; Strasburger, 2020; Strasburger et al., 2011). Here, we too use letters, because they do not require training and provide a large number of stimulus alternatives, which speeds threshold estimation in laboratory and clinical testing (Pelli, Robson, & Wilkins, 1988).

Whether crowding can be explained by the neural computations in any particular cortical location remains unknown, but several candidate areas have been suggested: V1 (Millin, Arman, Chung, & Tjan, 2014), V2 (Freeman & Simoncelli, 2011; He et al., 1996), V3 (Bi, Cai, Zhou, & Fang, 2009; Tyler & Likova, 2007), hV4 (Burchell, Benson, Zhou, Winawer, & Pelli, 2019; Liu et al., 2009; Motter, 2006; Zhou, Benson, Pelli, & Winawer, 2017), and higher-order areas (Aghdaee, 2005; Louie et al., 2007). The magnitude of the BOLD signal in V1 is lower in the presence of crowding (Millin et al., 2014). Crowding distance is different for stimuli tuned to stimulate either the parvo- or magnocellular pathway (Atilgan, Yu, & He, 2020). Although both crowding and acuity increase linearly with eccentricity, which might suggest a common physiological origin, the two lines have very different intercepts with the eccentricity axis; that is, the *E*_2_ value for acuity is more than 5 times larger than the *E*_2_ value for crowding (*E*_2_ = 2.72 for acuity; *E*_2_ = 0.45 for crowding) (Latham & Whitaker, 1996; Petrov & Meleshkevich, 2011; Rosenholtz, 2016; Song, Levi, & Pelli, 2014; Strasburger, 2020). This seems inconsistent with a common cause. Here, we use our data from 50 observers to study the relationship between acuity and crowding in the fovea.

In our 50 participants, we measured 13 crowding distances at three eccentricities (0, 5, and 10 deg) on all four cardinal meridians. We tested two crowding orientations (radial and tangential) and two fonts (Sloan and Pelli). We also measured acuity in the fovea using the Sloan and Pelli fonts. As far as we know, the Pelli font is still the only letter font skinny enough to measure crowding distance in the fovea. Apart from letters, foveal crowding can be measured with vernier targets (Malania et al., 2007). We also assessed the variation in crowding along the four cardinal meridians in two crowding orientations and across individuals. Crowding varies twofold across meridians, producing several asymmetries.

## Methods

### Measuring crowding

We measured thresholds of many participants, collecting two datasets with similar methods except for one important difference. Details about QUEST and stimulus presentation were similar for both and are described in the sections below. Here we focus on the differences.

#### Method 1: Unmonitored fixation

Threshold was measured without gaze tracking. Viewing distance was measured before each session, and no chin rest or forehead support was provided. The participant identified the target by pressing that letter in the keyboard. Participants were naive to the task and received no advance training. In each block, crowding distance was measured at two randomly interleaved target locations, which were horizontally or vertically symmetric about the fixation cross. This unmonitored-fixation dataset includes one radial crowding distance on each of the four cardinal meridians with the Sloan font for 100 participants.

#### Method 2: Awaited fixation

Each trial began only when the participant had continuously fixated within ±1.5 deg of the crosshair for 250 ms, and we only saved trials in which gaze remained within ±1.5 deg of the crosshair center until stimulus offset. The experimenter was present during data acquisition. Viewing distance was measured at the beginning of each session and maintained by use of a chinrest with forehead support. Participants identified the target by using a mouse to click on one of the letters displayed on the response screen. For each participant, data collection began after a total of 10 correct trials. Crowding distance was measured at four randomly interleaved target locations symmetric about the fixation cross, one on each cardinal meridian. We acquired two thresholds at each location to estimate test-retest reliability. This awaited-fixation dataset includes two radial crowding distances on each of the four cardinal meridians with the Sloan font for 50 participants.

#### Comparison of methods

**Figure 1.**
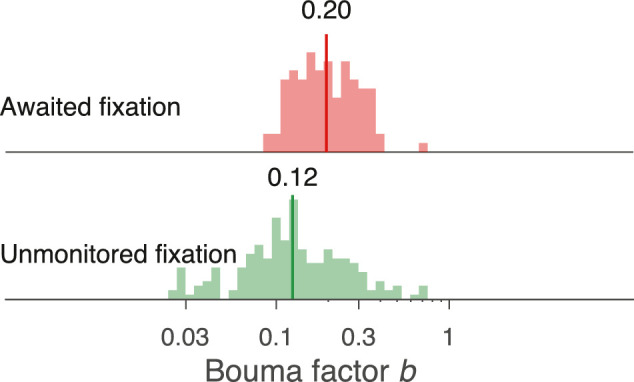
Histograms of the Bouma factor estimated by two methods with two possible target locations. Each histogram shows the Bouma factor *b* at ±5 deg eccentricity along the horizontal midline. For awaited fixation, we used only data from the first session of the experiment. The geometric mean *b* is indicated by a dark vertical line capped by a number. Unmonitored fixation gave a 0.12 geometric mean *b* with 0.31 *SD* of log *b*. Awaited fixation gave a higher 0.20 geometric mean *b* with a lower 0.18 *SD* of log *b* (see Table 3). The Results section reports a 0.78:1 right:left advantage. Note that mixing data from the two locations (−5 left and +5 deg right) makes the combined histogram slightly broader than that for either location. The 0.78:1 *b* ratio corresponds to a −0.107 log *b* difference. If we suppose that mixing log *b* estimates from the two locations is equivalent to taking all of the data from the right location and adding +0.107 to a random half of the log *b* estimates, then mixing the two locations increases the variance by +0.0025, which is only 8% of the measured variance of log *b* for awaited fixation, and only 3% for unmonitored fixation.

Comparing results obtained with the two methods at ±5 deg on the horizontal midline revealed large differences in the mean and distribution of the Bouma factor (Figure 1). (In this paper, “log” is the logarithm base 10.) Using unmonitored fixation, geometric mean *b* was 0.12, with a 0.31 standard deviation (*SD*) of log *b*. Using awaited fixation, geometric mean *b* was higher (0.20), with a lower *SD* of log *b* (0.18). The awaited-fixation histogram (red) is compact. The unmonitored fixation histogram (green) is much broader, extending to much lower values of *b*. Our interpretation of the broader histogram and lower geometric mean *b* in unmonitored fixation is that observers occasionally “peek”—that is, fixate near an anticipated location of the target instead of the fixation cross as instructed. Indeed, at the end of the Results section, we present a quantitative peeking model showing that peeking reduces geometric mean *b* and broadens its distribution, consistent with the observed results.

This paper focuses on the awaited-fixation data, which can be downloaded from the Center for Open Science (https://osf.io/83p6u/).

### Crowding dataset

Data were acquired using CriticalSpacing.m software (Pelli et al., 2016) with QUEST (Watson & Pelli, 1983), allowing for reliable and relatively fast measurement of crowding distance. Our crowding database consists of measurements of crowding distance with the Sloan font (with radial and tangential flankers) and with the Pelli font (radial flankers) in 50 observers. With the Sloan font, we measured crowding at eight different peripheral locations in the visual field: two eccentricities (5 and 10 deg) along the four cardinal meridians (upper, lower, left, and right). Sloan tangential crowding was measured only at ±5 deg eccentricity on the horizontal midline. With the Pelli font, we measured crowding at the fovea and at ±5 deg on the horizontal midline. The Sloan font acuity size is too big to allow measuring foveal crowding distance in adults. The Pelli font was specially designed for measuring foveal crowding distance (Pelli et al., 2016). We also measured acuity in the fovea. A spatial map of the testing is shown below (see Figure 4 in the Results section). We also tested 10 observers at 20 and 30 deg eccentricity with radial flankers (only one session; see Figure 9 for the plotted results). To estimate test–retest reliability of our measurements, we used two sessions to measure each threshold twice. Sessions were scheduled at least a day apart over a maximum of 5 days apart. We report our results as the Bouma factor *b* (slope of crowding distance vs. eccentricity) estimated from Equation 10, to minimize error in fitting log *ŝ*. Here, crowding distance *ŝ* is the required center-to-center spacing (in deg) for 70% correct report of the middle letter in a triplet.

### Participants

Table 1 describes our main dataset, and Figure 4 (in the Results section) plots its spatial coverage of the visual field. The study tested 50 observers (mean age = 23 years), mostly New York University undergraduate students. Each observer had normal or corrected-to-normal vision. All experiments were conducted in accordance with the tenets of the Declaration of Helsinki and were approved by New York University's ethics committee on activities involving human observers. In all analyses, except the test–retest section, we average the first- and second-session thresholds. The peeking-model section in the Results section refers to these “awaited-fixation” peripheral measurements on 50 participants and compares them to separate “unmonitored-fixation” peripheral measurements on 100 participants.

**Table 1.** Data summary. In the periphery, we measured crowding distance radially and tangentially with the Sloan font. With the Pelli font, we measured crowding both in the fovea and periphery. We also measured foveal acuity with the Sloan font. Each threshold was measured once in two sessions separated by at least 24 hours. For peripheral thresholds, we used gaze tracking to guarantee fixation within ±1.5 deg of the crosshair center. Foveal crowding requires a long viewwing distance which makes gaze tracking impractical, so participants were merely instructed to fixate the center of the crosshair. We suppose good fixation of the central crosshair because the participants expected a foveal target. *N* = 50.

Measure	Font	Crowding orientation	Radial eccentricity (deg)	Cardinal meridians	Thresholds per observer (each measured twice)	Gaze tracking
Crowding	Sloan	Radial	5, 10	All	8	Yes
Crowding	Sloan	Tangential	5	Right, left	2	Yes
Crowding	Pelli	Radial	5	Right, left	2	Yes
Crowding	Pelli	Horizontal	0	—	2	No
Acuity	Sloan	—	0	—	2	No

### Apparatus

Each testing session was completed on an iMac (Apple, Cupertino, CA) with a 27-inch external monitor. The observer viewed a 27-inch 5K monitor (27MD5KL-B; LG, Seoul, South Korea), with a screen resolution of 5120 × 2880 and a white background with luminance of 275 cd/m^2^. The white background never changed throughout the experiment; the black crosshair and letters were drawn on it. The observer viewed the screen binocularly at one of several different viewing distances. The software required a special keypress by the experimenter at the beginning of every block with a new observer or a new viewing distance to affirm that the new viewing distance (eye to screen) was correct as measured with a tape measure and that the screen center was orthogonal to the observer's line of sight. To measure crowding and acuity in the fovea, the viewing distance was 200 cm. For ±5 and ±10 deg eccentricity the distance was 40 cm, and for ±20 and ±30 deg eccentricity it was 20 cm. The long viewing distance gives good rendering of small stimuli; the short viewing distance results in a wide angular subtense of the display, which allows presentation of peripheral targets on either side of a central fixation. Stimuli were rendered using CriticalSpacing.m software (Pelli et al., 2016) implemented in MATLAB 2021 (MathWorks, Natick, MA) using the Psychtoolbox (Brainard, 1997; Pelli, 1997). Every Sloan letter was at least 8 pixels wide, and every Pelli digit was at least 4 pixels wide.

### Stimuli and procedure

**Figure 2.**
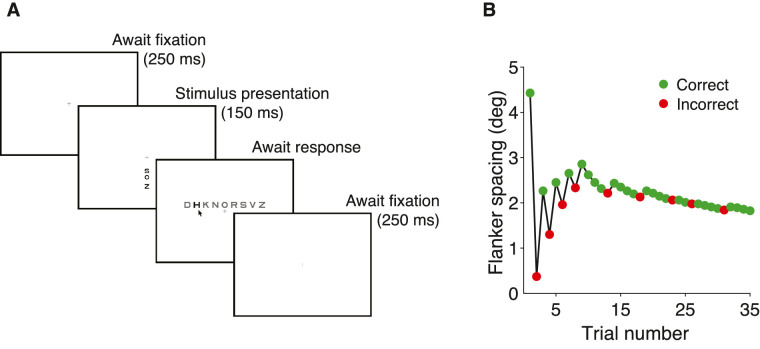
Stimulus and procedure. (**A**) The display sequence for a peripheral trial and part of the next. While gazing at the crosshair, which is always present, the observer presses the space bar, which begins the first trial. The target is presented once the observer had continuously fixated within 1.5 deg of the crosshair center for 250 ms. Stimulus presentation is accompanied by a low-pitched purr. Then the observer identifies the target by using a mouse to click on one letter out of all possible letters that appear above the fixation on the response screen. If the response was correct, the observer hears a brief beep acknowledging correctness and silence otherwise, then the computer again waits for 250 ms of fixation within 1.5 deg of the crosshair center. The four conditions (one for each meridian) are randomly interleaved, so the observer does not know which location comes next. (**B**) A QUEST staircase. The staircase sequence of spacings tested on 35 successive trials of one condition (+5 deg eccentricity), under control of QUEST. On each trial, the letter size was a fraction 1/1.4 of the spacing. QUEST picks the most informative spacing to test on each trial to minimize variance of its final threshold estimate. Finally, after 35 trials, QUEST estimated the crowding distance (i.e., spacing to achieve 70% correct). Notice that the testing quickly homed in on threshold.

To measure acuity, we showed one letter. To measure crowding, we showed a trigram of three letters or digits. For each trial, the three letters or digits were drawn randomly, without replacement, from the nine letters (DHKNORSVZ) or digits (123456789) available. Letters and digits were rendered as black in the Sloan or Pelli font and presented on a uniform white background (Pelli et al., 2016; Sloan, Rowland, & Altman, 1952). We omitted the C in the Sloan font because it is too easily confused with the O (Elliott, Whitaker, & Bonette, 1990). For crowding, each trigram was arranged either radially or tangentially. Each testing session included several blocks and was about an hour long. Most blocks measured four thresholds, interleaved, usually four crowding thresholds on the four cardinal meridians at the same radial eccentricity. For the Pelli font and tangential crowding, we measured two thresholds at symmetric locations about fixation along the horizontal midline. To minimize the temptation to look away from fixation toward an expected target location, we randomly interleaved conditions measuring threshold at the same radial eccentricity at two or four symmetric locations around fixation. A sample stimulus sequence appears in Figure 2A. A central crosshair (the fixation mark) was displayed until the observer pressed a key to initiate the trial. Then, after 250 ms of correct fixation, the letter trigram appeared on the screen for 150 ms and the computer waited for the observer to identify the middle stimulus letter using a mouse to click on a letter in a row of all the possible letters on the response screen. Observers were instructed to return their eyes to fixation before clicking their response. A correct response was acknowledged with a brief beep. The computer then waited indefinitely for the observer to fixate within 1.5 deg of the crosshair for 250 ms and then immediately presented the stimulus for the next trial. If the observer failed to fixate for 250 ms within a 10-second window, the software asked for recalibration of the gaze tracker.

### Measuring crowding distance

Crowding distance was estimated using the Pelli et al. (2016) procedure. In this paper, letter “size” is the width of the letter's bounding box. Letter spacing was controlled by QUEST. Letter spacing is proportional to letter size with a fixed ratio of 1.4:1. We set the Weibull function guessing rate parameter γ to the reciprocal of the number of characters in the test alphabet for that font, usually nine. We set the “finger error” probability δ to 1% to help QUEST cope with an occasional reporting mistake. We set the Weibull function steepness parameter β to 2.3, based on fits to two observers’ psychometric data for radial crowding. At the end of each block, QUEST estimated the threshold (crowding distance in deg) (Figure 2B). To measure acuity, we followed a similar procedure, except that the target was presented without flankers. Threshold was defined as the letter spacing (crowding distance, in deg) or letter size (acuity, in deg) that achieved 70% correct identification, using QUEST to control the stimulus parameters trial by trial and make the final estimate.

Each threshold measurement was based on 35 trials (one condition). A block consisted of all of the trials in however many conditions were randomly interleaved (e.g., 4 × 35 = 140 trials to measure the threshold at four meridians). Each condition measured the threshold for one meridian. The interleaving kept the observer uncertain as to which location was being tested on each trial. We did this to minimize the urge to “peek” away from fixation. On a crowding trial, until the target and flankers appeared, there was nothing about the display that distinguished which of the interleaved conditions that trial belonged to.

### Gaze tracking

We used gaze-contingent display to guarantee fixation while measuring all peripheral thresholds. We used an EyeLink 1000 eye tracker (SR Research, Ottawa, ON, Canada) with a 1000-Hz sampling rate. To allow short viewing distance (40 cm) we used the EyeLink Tower mount with a 25-mm lens mounted on the EyeLink camera. Each trial presented the stimulus when gaze had been within 1.5 deg of the crosshair center (the fixation mark) for 250 ms. If, during the stimulus presentation, gaze deviated more than 1.5 deg from the crosshair center, then the trial was not saved, the fixation cross turned red (to alert the participant), and the trial was repeated with a fresh letter trigram. Thus, each threshold estimate was based on 35 trials with fixation within 1.5 deg of the crosshair center. The foveal thresholds demanded a long 200-cm viewing distance that was incompatible with our gaze tracking set-up, so they were measured without gaze tracking, but fixation is generally good when the participant knows that the target is foveal.

### Model fitting

It is generally found that the *SD* of repeated measurements of threshold spacing *s* is roughly proportional to the mean spacing, but the *SD* of log spacing *S* is independent of mean spacing. Therefore, our fitting minimizes the root-mean-square (RMS) error in log spacing *S *= log_10_*s*. The fitting is nonlinear (using the MATLAB fmincon function) because we minimize error in *S*, whereas each model is linear in *s*, not *S*. We estimated the participant, meridional, crowding orientation, and font factors by solving several models (see model equations in Table 4).

Our fitting minimizes the RMS error in predicting the log crowding distances, which is equivalent to minimizing the summed square error (*SSE*):

(1) $$\begin{array}{c} SSE=\sum_{i}{\left(S_{i}-{\hat{S}}_{i}\right)}^{2}\; \end{array}$$

where *S_i_* is the *i*-th log crowding distance, and ${\hat{S}}_{i}$ is the *i*-th predicted log crowding distance. The variance explained by each model is

(2) $$\begin{array}{c} R^{2}=1-\sum_{i}{\left(S_{i}-{\hat{S}}_{i}\right)}^{2}/\;\sum_{i}{\left(S_{i}-\bar{S}\right)}^{2}\; \end{array}$$

where $\bar{S}$ = mean*_i_* *S_i_* is the mean log crowding distance.

### Model comparison

An *F* test was used for pairwise model comparison. The model with fewer parameters is referred to as “simple,” and the model with more parameters is referred to as “full.” After calculating the sum of squared errors *SSE*_simple_ and *SSE*_full_ for each model (Equation 1), we calculated the *F*-statistic:

(3) $$\begin{array}{c} F=\frac{\left(SSE_{\mathrm{simple}}-SSE_{\mathrm{full}}\right)/\left(n_{\mathrm{full}}-n_{\mathrm{simple}}\right)}{SSE_{\mathrm{full}}/\left(N-n_{\mathrm{full}}\right)}\; \end{array}$$

where *n*_full_ is the number of parameters in the full model, *n*_simple_ is the number of parameters in the simple model, and *N* is the number of observations. The *p* value is estimated using the *F* distribution. A *p* value less than 0.05 indicates that the model with more parameters provides a significantly better explanation of the data.

### Cross validation

First, we divided the thresholds into six random subsets of equal size. In each cross-validation step, one subset of data was retained as the validation set for testing the model, and the remaining subsets were used as training data. Each subset was chosen only once for testing. We repeated leave-one-out testing six times to obtain the full dataset. Variance explained *R^2^* is calculated by Equation 2.

### Standardized Bouma factor can be compared across studies

To facilitate comparison across studies and the cooperative evaluation of the Bouma factor as a biomarker of visual health, we define the standardized Bouma factor *b*′ as the slope of crowding distance versus radial eccentricity multiplied by a correction factor that accounts for methodological differences from Bouma's number of choices (25), threshold criterion (75% correct), and linear spacing (vs. log).

Table 2 computes the correction factors needed to compare the Bouma factor *b* across studies that used various numbers of response choices (e.g., nine Sloan letters or two orientations of a tumbling T), various threshold criteria (e.g., 70% or 75% correct), and linear or log flanker spacing. Including the present one, we know of five studies that have compared crowding distance across meridians. We have taken Bouma (1970) as the standard for this standardized way of reporting the strength of crowding.

**Table 2.** Correction factor to standardize the Bouma factor of each study to compare studies. The resulting standardized Bouma factors are reported in Table 6. The differences in each study from Bouma's 25 choice alternatives and 75% correct threshold criterion offset the threshold log spacing by *∆S* relative to Bouma's. The correction factor is 10^–*∆S*. We provide a brief Excel spreadsheet to calculate the correction factor and a MATLAB script that produces plots like Figure 3. The correction factor to account for linear versus log flanker spacing is estimated in the Supplementary Materials. Note that crowding measured with tangential flankers does not require correction for log versus linear spacing symmetry. Tangential spacing was always linear.

Study	Target	Eccentricity (deg)	Choices, *n*	Guessing rate, γ = 1/*n*	Threshold criterion proportion correct *P*	Threshold criterion “true” proportion correct, *p* (Equation 5)	Log threshold shift *∆S* = *S* – *S*_Bouma_ (Equation 9)	Correction factor to standardize Bouma factor for number of choices and the threshold criterion (10^–*∆S*)	Correction factor to standardize Bouma factor for log vs. linear flanker spacing (1.18 for log,1 for linear)
Bouma (1970)	Courier 10, letter	1–7	25	1/25	0.75	0.74	0.00	1.00	1
Our data	Sloan or Pelli, letter	5,10	9	1/9	0.70	0.66	−0.04	1.10	1.18
Greenwood et al. (2017)	Tumbling clock	4,8	4	1/4	0.80	0.73	0.00	1.01	1
Toet & Levi (1992)	Tumbling T	2.5, 5 ,10	2	1/2	0.75	0.50	−0.13	1.33	1
Grainger et al. (2010)	Courier New, letter or symbol	3	9	1/9	0.75	0.72	−0.01	1.03	1
Coates et al. (2021)	Tumbling T	9	4	1/4	0.625	0.50	−0.13	1.33	1

**Figure 3.**
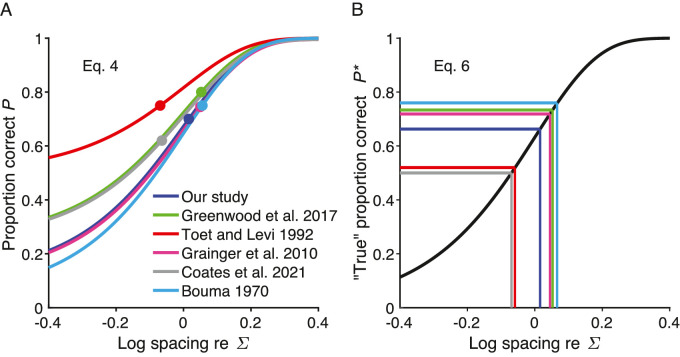
Effect of guessing rate and criterion on threshold log crowding distance *S*. (**A**) Proportion correct (Equation 4) of the six studies, with threshold parameter Σ set to zero, showing the effects of the number of response choices *n* (sets lower asymptote γ = 1/*n*) and the threshold criterion (height of each colored dot). (**B**) Psychometric function corrected for guessing (Equation 6) for the same studies. Each study's threshold criterion P* is represented by a horizontal line. For each study, a vertical line reads off the log threshold spacing *S* at its threshold criterion P*. Results from Bouma (1970) are used with the Andriessen and Bouma (1976) 75% threshold criterion. Table 2 computes the difference between each study's threshold and Bouma's. To avoid occlusion, the Toet and Levi and Bouma lines in this panel were offset by +0.02 horizontally and vertically.

The log-threshold shift *∆S* is illustrated in Figure 3B. Using the Coates et al. (2021) reanalysis of Bouma's (1970) data, we estimated the Bouma factor for a 75% threshold criterion applied to Bouma's (1970) results (see the Bouma factor paragraph in the Discussion section).

#### Proportion correct

To account for the different number of choices and the threshold criterion, we assumed that, with accurate fixation, the proportion correct *P* is a Weibull function of log spacing *S*:

(4) $$\begin{array}{c} P\left(S\right)=\gamma +\left(1-\gamma \right)\left[1-\exp\left(-10^{\beta \left(S-\Sigma \right)}\right)\right]\; \end{array}$$

with a threshold parameter Σ, where γ is the guessing rate (the reciprocal of the number of target choices, which is 1/25 = 0.04 in Bouma's 1970 results), and β is the steepness parameter, which we set to 2.3, based on fitting psychometric functions to hundreds of trials at several spacings by two experienced observers. Figure 3A shows this psychometric function for six studies, taking the guessing rate γ to be the reciprocal of the number of choices *n*, and using our own estimate of the steepness parameter β = 2.3.

#### “True” proportion correct

To accommodate various numbers of choices *n*, and thus guessing rates γ = 1/*n*, we corrected for guessing:

(5) $$\begin{array}{c} P^{*}=\left(P-\gamma \right)/\left(1-\gamma \right)\; \end{array}$$

This is a popular transformation of psychometric data, usually justified by assuming that the guessing rate can be modeled as an independent process. Because it discounts false alarms, the corrected hit rate is referred to as the “true hit rate.” That makes sense for a yes/no task, but not for an identification task. Here we proceed regardless and compute the “true” proportion correct, because, with this Weibull function (Equation 4), correction for guessing (Equation 5) removes all dependence on γ. Applying correction for guessing to any given threshold criterion *P* gives us the corresponding “true” proportion correct criterion P^*^ to apply after correction for guessing. Similarly, applying correction for guessing to the psychometric function (Equation 4) gives us the “true” proportion correct:

(6) $$\begin{array}{c} P^{*}\left(S\right)=1-\;\exp\left(-10^{\beta \left(S-\Sigma \right)}\right)\; \end{array}$$

The inverse of Equation 6 is

(7) $$\begin{array}{c} S=\mathrm{inv}P^{*}\left(P^{*}\right)=\;\Sigma +\log\left[-\ln\left(1-\;P^{*}\right)\right]/\beta \; \end{array}$$

Figure 3B plots the “true” proportion correct (Equation 6 with β = 2.3 and Σ = 0), the same function for all studies, and, for each study, a vertical line reads off the log threshold spacing *S* at its threshold criterion P^*^.

#### Relative to the Bouma standard

Thus, the number of choices of a study and the threshold criterion increase its log threshold by ∆*S* relative to the Bouma standard:

(8) $$\begin{array}{c} \Delta S=\mathrm{inv}P^{*}\left(P^{*}\right)-\mathrm{inv}P^{*}\left(P_{\mathrm{Bouma}}^{*}\right)\; \end{array}$$

(9) ΔS=log-ln1-P*/βΔS=-log(-ln1-PBouma*)/β

where β = 2.3, and P^*^ and P Bouma * are the “true” proportion correct threshold criteria computed by Equation 5 from the study's criterion *P* and Bouma's *P*_Bouma_ = 0.75 (Andriessen & Bouma, 1976).

### Log-symmetric spacing of flankers

Since the study by Bouma (1970), most crowding studies have measured crowding distance as the center-to-center spacing between the target and each of two flankers on opposite sides of the target that yields a criterion level of performance. When crowding is measured in the radial orientation, the Bouma law tells us that crowding distance increases linearly with eccentricity. Several studies have documented that, when flankers are arranged symmetrically about the target on a radial line from fixation, the outer flanker has much more effect (Banks, Bachrach, & Larson, 1977; Bex & Dakin, 2005; Estes, Allmeyer, & Reder, 1976; Krumhansl, 1977). This is to be expected because crowding distance grows with eccentricity and the outer flanker is more eccentric. In fact, crowding distance on the cortical surface (in mm)—the product of crowding distance (in deg) and cortical magnification (in mm/deg)—is conserved across eccentricity (for eccentricities above 5 deg) because psychophysical crowding distance scales with eccentricity (Bouma, 1970; Kooi et al., 1994; Levi & Carney, 2009; Pelli et al., 2004; Toet & Levi, 1992). Given the logarithmic cortical mapping of the visual field (Fischer, 1973), when measuring radial crowding we space the trigram so that the log eccentricity of the target is midway between the log eccentricities of the flankers and report the inner spacing. This raises the question of how to compare crowding distances among experiments that have spaced the flankers linearly versus logarithmically. Given the Bouma law (Equation 10 below), supposing that crowding distance depends primarily on the flanker-to-flanker distance and only negligibly on the target position between them, we show in the Supplementary Materials (“Effect of symmetric placement of flankers with regard to either linear or log eccentricity”) that the crowding distance is expected to be 1.18 times larger when measured with linearly spaced flankers than with log-spaced flankers. To ease comparison across studies, the correction factors in Table 2 include this effect of log versus linear spacing on the estimated Bouma factor.

### Might attention help explain differences in the reported Bouma factor?

Attention reduces many perceptual thresholds (Carrasco, 2011). Many researchers have assessed the effects of attention on crowding, but they have yet to reach a consensus. Several have found an attentional benefit in crowding tasks (Bacigalupo & Luck, 2015; Kewan-Khalayly, Migo, & Yashar, 2022), including reductions of crowding distance (Yeshurun & Rashal, 2010), but others have not found such effects (Scolari, Kohnen, Barton, & Awh, 2007; Strasburger, 2005; Strasburger & Malania, 2013). All of our peripheral crowding thresholds were measured with either twofold or fourfold uncertainty about target location, and we supposed that attention was distributed among the possible target locations. It is possible that attentional bias contributed to some of the Bouma factor asymmetries.

### Data from other studies

Data were extracted from Figure 6 of Toet and Levi (1992) using WebPlotDigitizer (Rohatgi, 2022). Data were extracted from Figure 7 of Grainger et al. (2010). Data from Greenwood et al. (2017, Supplementary Figure S1) and Coates et al. (2021, Figure 10) were received as personal communications from the authors. Data from Bouma (1970) were used by means of the recent reanalysis by Coates et al. (2021).

### Statistical analysis

Statistics of the log Bouma factor *B* = log *b* were assessed using an analysis of variance (ANOVA) with *B* as the dependent variable. Two sample comparisons were made with the Wilcoxon rank-sum test. We report Pearson's *r* correlation coefficient for test–retest reliability and correlations of crowding distance.

## Results

### Crowding and acuity

**Figure 4.**
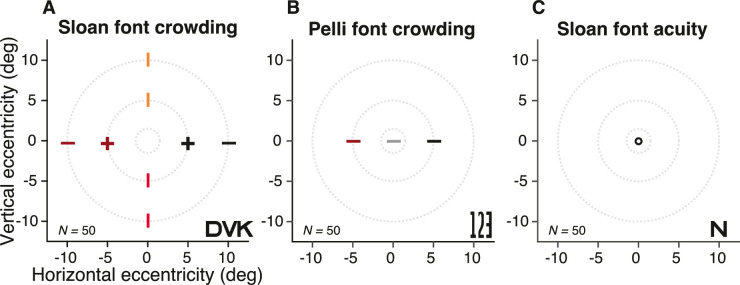
Map of testing. Each panel title indicates the font and threshold task. The number of observers tested is indicated in the lower left. The +, −, and o symbols indicate testing of crowding with radial (−) or radial and tangential (+) flankers, and testing of acuity (o). Typical stimuli appear in the lower right of each panel. Beyond the main dataset described here, Figure 9 shows additional results from 10 observers at 20 and 30 deg eccentricity.

As shown in the map of testing (Figure 4), radial crowding thresholds were measured in 50 adults at nine visual field locations. Using the Sloan font, radial crowding thresholds were measured at the four cardinal meridians at 5 and 10 deg eccentricity, and tangential crowding thresholds were measured on the left and right meridians at 5 deg eccentricity. Using the Pelli font, the horizontal crowding threshold was measured in the fovea and on the right and left meridians at 5 deg eccentricity. Foveal acuity was also measured with the Sloan font.

### Test–retest reliability of the visual threshold

**Figure 5.**
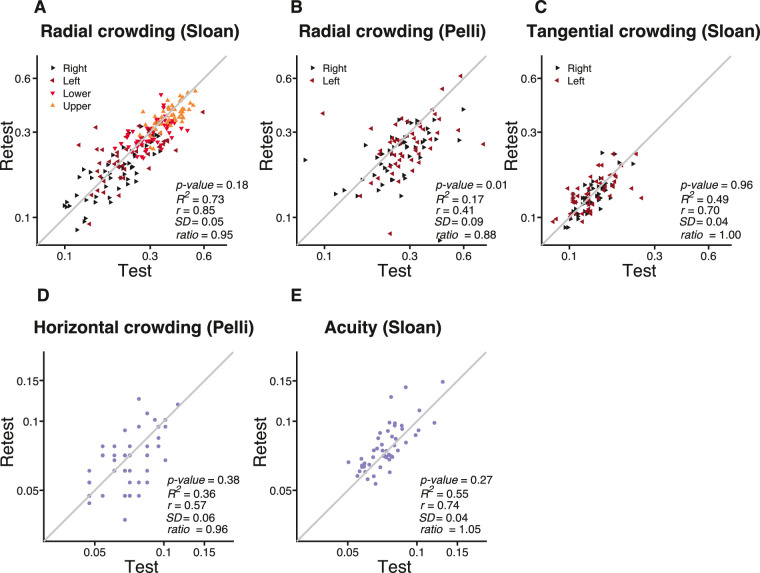
Test–retest reliability of threshold estimates. Estimates of Pearson's *r* correlation coefficient, *SD*, retest:test ratio, and *R*^2^ are based on log Bouma factor, horizontal spacing, or acuity named at the top of each panel. For peripheral crowding (**A**–**C**) each measurement is represented by a triangle pointing toward the tested meridian. The gray line represents equality. In each panel, *SD* represents the mean across observers of the *SD* of test and retest of log *b*. “Ratio” is the geometric mean across participants of each participant's ratio of retest *b* over test *b*. The *p* value is from a two-sample *t*-test between test and retest and *r* is Pearson's correlation coefficient.

Measurement reliability was assessed by measuring each threshold twice, at least 1 and not more than 5 days apart. Crowding thresholds are converted to Bouma factors *b* (see Equation 10 below). Foveal crowding and acuity are presented as crowding distance (deg) and acuity as letter size (deg).  Figure 5 plots a scatter diagram of estimates from first versus second session for each combination of font and task. The second session improved over the first only for the Pelli font (Figure 5B), with a ratio of geometric mean retest:retest = 0.88. This training benefit was much smaller (and insignificant) for the Sloan font (0.95), presumably because Sloan is more similar (than Pelli) to familiar fonts and thus benefits less from learning. In general, each threshold is derived from a QUEST staircase with 35 trials, which takes about 3.5 minutes and has very good reproducibility. The analyses performed in the following sections are based on the geometric mean threshold across both sessions.

### Analysis of variance

Table 3 presents an ANOVA analysis of the radial Sloan Bouma factors (also plotted in Figure 5A). The 0.18 *SD* for radial Sloan with two meridians (with awaited fixation) in Figure 1 corresponds to the 0.17 total *SD* with four meridians (also with awaited fixation) in Table 3. The 0.08 *SD* for Sloan radial crowding in Figure 8 corresponds to the 0.08 *SD* across observers in Table 3. Meridian contributed the most variance.

**Table 3.** Analysis of variance. We computed the contribution of each parameter to overall variance. Meridian contributed the most (*SD* = 0.12) and test–retest contributed the least (*SD* = 0.01). The degrees of freedom values are the number of parameters minus 1. Error is the remaining variance not accounted for by a linear combination of meridian, observer, and test–retest. There were no significant pairwise interactions among meridian, test–retest, and observer (all *p* > 0.5).

Factor	Degrees of freedom	Variance	SD	*p*
Meridian	3	0.0143	0.1195	<0.01
Observer	49	0.0059	0.0770	<0.01
Test–retest	1	0.0001	0.0109	0.016
Error	346	0.0071	0.0843	—
Total	399	0.0274	0.1656	—

### Bouma law explained Bouma law explained 82% of variance

Bouma (1970) discovered the linear relationship between crowding distance and eccentricity. He initially reported a slope of 0.5, which he later revised to 0.4 (Andriessen & Bouma, 1976). The Bouma law is

(10) $$\begin{array}{c} \hat{s}\;=\left(\varphi_{0}+\varphi \right)b\; \end{array}$$

where *ŝ* is crowding distance (in deg), φ is radial eccentricity (in deg), and φ_0_ (in deg) and *b* (dimensionless) are positive fitted constants (Bouma, 1970; Rosen, Chakravarthi, & Pelli, 2014). The dimensionless slope *b* is the Bouma factor. The horizontal intercept is –φ_0_, and the vertical intercept is φ_0_*b* (Liu & Arditi, 2000; Strasburger et al., 2011; Toet & Levi, 1992).

Crowding is one of several tasks for which the threshold increases linearly with radial eccentricity, and such a task can be summarized by an *E*_2_ value that is the eccentricity at which threshold reaches twice its foveal value (Levi et al., 1985). In the Bouma law (Equation 10), *E*_2_ = φ_0._

Our large database of visual crowding thresholds (Table 1) is very well fit (*R*^2^ = 82.45%) by the two-parameter linear Bouma law (Equation 10), showing that most of variation in crowding in our data is explained by eccentricity. Just two degrees of freedom, *b* and φ_0_, suffice to fit all 650 data points (13 thresholds measured in each of 50 observers). The estimated slope *b* was 0.23, just over half of Bouma's 0.4. (We return to this apparent discrepancy below in Discussion: Standardized Bouma factor.) Our database consists of measurements at five locations with radial and tangential flankers and two fonts. To capture the effect of these parameters on the Bouma factor we propose an extended version of the Bouma law.

### Extended Bouma law explains 94% of variance

Crowding depends on more than just eccentricity. Crowding varies substantially across meridians (right, left, up, or down), crowding orientation (radial or tangential), target kind (e.g., letters or symbols) and across individuals. Here, we enhance the Bouma law by including these other variables. One by one, the extensions add model parameters for meridian, crowding orientation, target kind, and observer. The models and the variance that they account for are summarized in Table 4.

**Table 4.** How well the Bouma law and its extensions predict crowding distance. We began with the Bouma law (Equation 10). Successive models then tried to account for more variance by adding factors that depend on the meridian, crowding orientation, font, and observer. Each row gives a significantly better fit than the row above (assessed with *F*-test using Equation 3). The *R*^2^ (Equation 2) column shows cross-validated variance accounted for in predicting log crowding distance over the whole visual field (13 thresholds per observer). Pearson's *R* shows the correlation between acquired and predicted data, and RMSE is the root-mean-square error.

Model	Equation	*R^2^*	*R*	RMSE	No. of parameters	Deg. of f.
Bouma law	*ŝ* = (φ_0_ *+* φ)*b* (Equation 10)	82.45%	0.90	4.80	2	2
× Meridional factor	*ŝ* = φ_0_*b +* φ*b*_θ_ (Equation 11)	88.96%	0.94	3.80	6	5
× Crowding orientation	*ŝ* = (φ_0_*b +* φ*b*_θ_) *f*_dir_ (Equation 12)	92.54%	0.96	3.13	8	6
× Target kind factor	*ŝ* = (φ_0_*b +* φ*b*_θ_) *f*_dir_ *t*_kind_ (Equation 13)	92.63%	0.96	3.11	10	7
× Observer factor	*ŝ* = (φ_0_*b +* φ*b*_θ_) *f*_dir_ *t*_kind_ *o_i_* (Equation 14)	93.86%	0.97	2.84	60	57

#### Meridian

Factor *b*_θ_, which allows *b* to depend on the meridian θ (right, left, up, or down):

(11) $$\begin{array}{c} \hat{s}=\varphi_{0}b+\varphi b_{\theta }\;\; \end{array}$$

where *b* from Equation 10 now represents the *geometric mean* of *b*_θ_, *b* = $10\hat{\;}\left(\mathrm{mean}\left(\log b_{\theta }\;\right)\right)$. Note that the meridian is undefined at the fovea.

#### Crowding orientation

Factor *f*_dir_ depends on crowding orientation (radial or tangential):

(12) $$\begin{array}{c} \hat{s}=\left(\varphi_{0}b+\varphi b_{\theta }\right)f_{\mathrm{dir}}\; \end{array}$$

#### Target kind

Factor *t*_kind_ depends on target kind (e.g., Pelli or Sloan font):

(13) $$\begin{array}{c} \hat{s}=\left(\varphi_{0}b+\varphi b_{\theta }\right)f_{\mathrm{dir}}t_{\mathrm{kind}}\; \end{array}$$

#### Observer

Finally, factor *o_i_* depends on the observer:

(14) $$\begin{array}{c} \hat{s}=\left(\varphi_{0}b+\varphi b_{\theta }\right)f_{\mathrm{dir}}t_{\mathrm{kind}}o_{i}\; \end{array}$$

where $\prod_{i}o_{i}=1$. Adding factors to the original Bouma law accounts for more variance. Going from the simplest to the most enhanced model (Equations 10 and 14) increases explained variance from 82% to 94% (Table 4). Model performance is improved by adding the meridian factor (*R*^2^ = 89%) and crowding orientation (*R*^2^ = 93%). Adding the target-kind factor explains hardly any more variance, with an increase from 92.54% to 92.63%. Finally, the most enhanced model, with an observer factor, explains 94% of variance. The models are all cross-validated, so the additional variance explained is not a necessary consequence of the increase in parameters. If the additional parameters were overfitting the training data, then we would find less variance accounted for in the left-out test data.

Because model parameters can be added in any order to form the final model (Equation 14), we asked how much each parameter contributes to the total explained variance. For each parameter, we began with the full model, removed that parameter, calculated the explained variance for the reduced model, and assessed the drop in explained variance. We found that, after eccentricity, meridian contributed the most (4.5%) and target kind contributed the least (0.2%). (Note that most of our data were collected with just one crowding orientation and one font; we expect target kind to explain more variance in studies that more evenly use the two crowding orientations or several fonts, or other target kinds.) Results are shown in Table 5.

**Table 5.** Parameter contribution to the most extended Bouma law. We measured the contribution of each parameter to the full model.

Removed factor	Equation	Decrease in *R*^2^
Meridian	*ŝ* = (φ_0_ *+* φ) *b f_dir_* *t_kind_ o_i_*	4.5%
Crowding orientation	*ŝ* = (φ_0_*b +* φ*b*_θ_) *t_kind_* *o_i_*	3.1%
Observer	*ŝ* = (φ_0_*b +* φ*b*_θ_) *f_dir_* *t_kind_*	1.4%
Target kind	*ŝ* = (φ_0_*b +* φ*b*_θ_) *f_dir_* *o_i_*	0.2%

**Figure 6.**
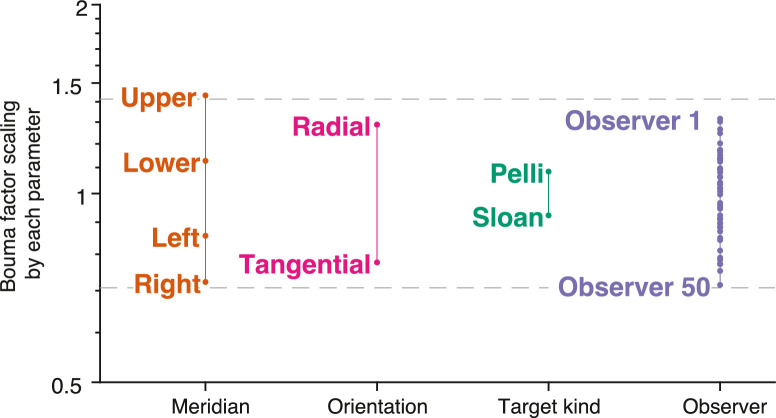
How several parameters scale Bouma factor. To reveal the effect of each parameter (horizontal axis) each set of model parameters was normalized by the geometric mean of that set. The vertical axis plots the model's estimates of that parameter. Because the model is multiplicative, the final Bouma factor is proportional to the product of all of the parameters. We found a similar variation of the Bouma factor with meridian, crowding orientation, and observer. Target kind had the least effect, but that is partly because nearly all of our data are with one font.

Because the enhanced model accounts for more variance than the original Bouma law, we looked in the data for systematic effects of these parameters. Figure 6 plots each set of model parameters after normalizing by the geometric mean of that set, where a set is the four meridians, two crowding orientations, two fonts, or 50 observers. Asymmetry within each factor is discussed in the next section. Except for target kind, each of the factors (meridian, crowding orientation, and observer) accounts for a roughly twofold variation in the Bouma factor (dashed horizontal lines in Figure 6).

### Radial Bouma factor varies twofold across meridians

**Figure 7.**
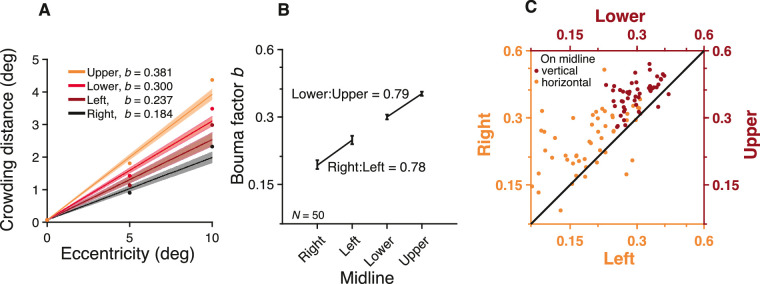
Bouma factor versus meridian. (**A**) Bouma law estimates for radial crowding with the Sloan font estimated using Equation 10. Each point represents the mean across participants, and error bars represent 95% confidence intervals. (**B**) Bouma factor versus meridian. (**C**) Individual participant data plotted for the vertical (dark red) and horizontal midline (orange).

Most of the thresholds in our dataset are for the Sloan font with radial flankers. Using these data, we explored the variation of the Bouma factor across meridians. We estimated the Bouma factor by fitting Equation 10 for each participant and meridian independently (Figure 7A). The Bouma factor was smallest along the right meridian and highest along the upper meridian (Figure 7B). The Bouma factor was 0.184 right, 0.237 left, 0.300 lower, and 0.381 upper meridian, with an overall geometric mean of 0.27.

#### Meridional asymmetries

We found three asymmetries. As a reporting convention, we refer to the “advantage” of a smaller Bouma factor. (1) Along the vertical midline, there is a 0.79 lower:upper advantage. (2) Along the horizontal midline, there is a 0.78 right:left advantage. (3) Finally, there is a 0.62 horizontal:vertical advantage (based on the geometric mean of the Bouma factors from the right and left meridians vs. upper and lower meridians). All reported asymmetries were highly consistent across participants (Figure 7C). ANOVA revealed a significant effect of meridian, *F*(3,196) = 92.76, *p* < 0.001, and post hoc analysis showed that the Bouma estimates at each meridian were significantly different from each other (all *p* < 0.001, corrected for multiple comparisons). For each meridian, we also estimated the eccentricity φ_0_ at which the crowding distance reached twice its foveal value; φ_0_ was 0.37 ± 0.02 deg for the right, 0.29 ± 0.02 deg for the left, 0.22 ± 0.01 deg for the lower, and 0.17 ± 0.01 deg for the upper meridians.

### Tangential Bouma factor is roughly half of radial

Unlike radial crowding, tangential crowding was the same in the left and right meridians according to the Wilcoxon rank sum test (*z* = −0.73, *p* = 0.49). The standardized (see section on corrected Bouma factor below) tangential Bouma factor was small: 0.13 on the right and 0.14 on the left meridian. The tangential:radial ratio was 0.60 in the right meridian and 0.50 in the left.

### Bouma factor varies with target kind

Pelli and Tillman (2008) highlighted the remarkable degree to which crowding distance is conserved across stimulus kind, but later work shows that crowding distance does differ substantially between some target kinds (e.g., letters vs. symbols) (Grainger et al., 2010). Along the horizontal midline, the standardized Bouma factor for the Sloan font was 0.239 on the right and 0.308 on the left, and it was slightly higher for the Pelli font: 0.325 on the right and 0.377 on the left. Overall, there was a 0.78 Sloan:Pelli ratio of standardized Bouma factors (geometric mean of the ratio taken at each meridian), and the difference between fonts was statistically significant (*z* = 3.58, *p* < 0.001). The model performance is slightly improved by adding the target-kind factor (Equation 13). This factor contributed little to the overall variance explained by the model because most of the data came from trials with the same target kind (Sloan letters). So, even though excluding target kind as a factor from the model caused inaccurate predictions for the Pelli font, the reduction in variance explained was negligible because nearly all of the dataset is based on the Sloan font. We anticipate that the target-kind factor will account for more variance in datasets that focus on comparing target kinds.

### Bouma factor varies twofold across observers

**Figure 8.**
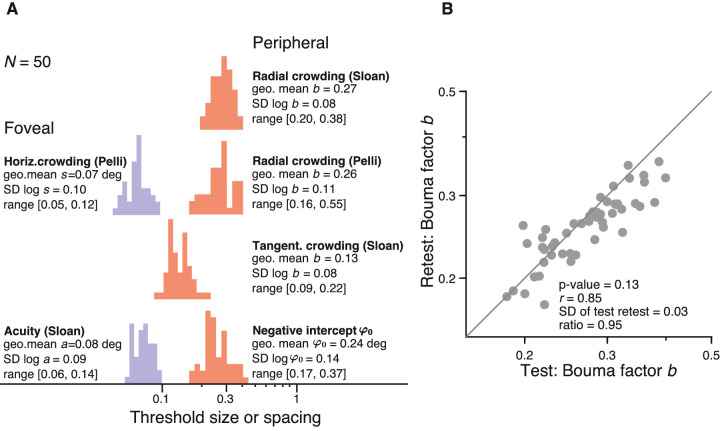
Histograms of crowding and acuity. Histograms of (**A**) radial Sloan, radial Pelli, and tangential Sloan Bouma factor (dimensionless), and radial Sloan negative intercept φ_0_ (in deg), foveal horizontal crowding distance *s* (in deg), and foveal acuity *a* (in deg). To estimate individual differences, we used all data (i.e., in the periphery: 16 radial crowding thresholds with Sloan font, four radial crowding thresholds with Pelli font, and four tangential crowding thresholds with Sloan font; in the fovea: two horizontal crowding thresholds with Pelli font and two size thresholds with Sloan font). (**B**) Retest versus test of the Bouma factor *b* for radial Sloan, one point per observer. Separately for test and retest, the *b* estimate is the geometric mean across four meridians of the Bouma factor fitted to the observer's spacing thresholds at 5 and 10 deg. Listed parameters are calculated as in Figure 5.

The Bouma factor varied with meridian, crowding orientation, and target kind. Here, in this section, we quantify differences among observers. First, we estimated how well the Bouma law fits individual participant data. Fitting Equation 10 to the right meridian data for each participant resulted in, on average, 97% explained variance, confirming that individual crowding data are well described by the linear model. Next, for each observer, we fit the whole model to estimate the observer's overall Bouma factor (Figure 8). We also reported individual differences in acuity. Individual differences are characterized by the *SD* of the log of the threshold. The radial Bouma factor for the Sloan font varied approximately twofold across observers (*SD* of log *b* = 0.08). The variance was very similar for tangential flankers (*SD* of log *b* = 0.08) and larger for the Pelli font (*SD* of log *b* = 0.11). Foveal acuity *a* and foveal crowding distance *s* also varied twofold. For crowding, the φ_0_ values also varied twofold and ranged between 0.17 and 0.37 (Song et al., 2014). We also report the SD between test and retest for the log Bouma factor estimated with radial flankers and the Sloan font (Figure 8B). For each observer, we fit one log Bouma factor for the test session and one log Bouma factor for the retest. Differences across observers were much larger than those of test–retest. The 0.08 *SD* of the log Bouma factor across observers is nearly three times larger than the 0.03 *SD* of test and retest, showing that one such Bouma factor estimate, measured in half an hour, is enough to distinguish individual differences. That measurement of log *b* consists of eight thresholds (2 eccentricities × 4 meridians) and 280 trials (8 thresholds × 35 trials/threshold).

### Supralinearity: Bouma factor increases with eccentricity

**Figure 9.**
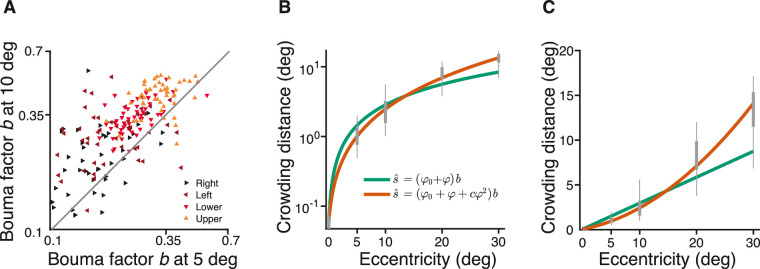
Supralinearity: Crowding distance slope grows with eccentricity. (**A**) Radial Bouma factor at 5 versus 10 deg eccentricity for the Sloan font. Each color shows data on a different meridian. Data are plotted for all 50 participants included in the main study. (**B**) Log crowding distance for 10 participants is plotted against eccentricity out to 30 deg. The linear Bouma law is green, and the quadratic Bouma law is orange. We used log coordinates because the fit minimizes the error in log coordinates. Enhancing the Bouma law from linear to quadratic increases the explained variance from 90% to 95%. (**C**) Same fits replotted in linear coordinates. The nonlinear growth of crowding distance with eccentricity is not an artifact of perspective transformation: The computation of target angular size and eccentricity was done correctly using the arc tangent function. Equation 10 fit with RMS error = 0.20, *b =* 0.30, and φ_0_ = 0.20. Equation 15 fit with RMS error = 0.16, *b =* 0.15, φ_0_ = 0.43, and *c* = 0.06.

Bouma discovered the linear increase of crowding distance with eccentricity. We have seen that this linear equation fits our data well. However, seeing that we had 50 participants and data at 0 to 10 deg, a reviewer suggested that we examine how well Bouma factor is conserved across eccentricity. To estimate the Bouma factor, we fit Equation 10 for the Sloan font with radial flankers to our data at 0 deg plus either 5 or 10 deg (Figure 9A). On average, the Bouma factor was 1.4 higher at 10 than 5 deg eccentricity. This effect was statistically significant, *F*(1,398) = 42.3, *p* < 0.001, and there was no interaction between eccentricity and meridian, *F*(3,392) = 0.513, *p* = 0.674. This shows that the growth of crowding distance with eccentricity is actually more than linear. Indeed, the Coates et al. (2021) reanalysis of Bouma (1970) shows a similar supralinearity. Motivated by this finding, we invited 10 observers already in the main dataset (0, 5, and 10 deg) to also measure crowding distance at 20 and 30 deg eccentricity.

When fitting data, there is a long tradition of using the shortest polynomial that fits adequately. Bouma (1970) initially suggested proportionality, with 1 degree of freedom. Measurements of nonzero crowding distance at 0 deg eccentricity led to a linear equation with 2 degrees of freedom. Seeing curvature in our data from 10 observers from 0 to 30 deg, we enhanced Bouma law from linear to quadratic (3 degrees of freedom) to fit the data. Equation 15 adds a quadratic term to Equation 7 to allow the slope to grow with eccentricity. Replacing Equation 10 by 15 increased the degrees of freedom from 2 to 3 and increased the explained variance from 90% to 95% (Figures 9B, 9C):

(15) $$\begin{array}{c} \hat{s}\;=\left(\varphi_{0}+\varphi +c\varphi^{2}\right)b\; \end{array}$$

where $\hat{s}$ is predicted crowding distance (in deg), φ is radial eccentricity (in deg), and φ_0_ (in deg), *b*, and *c* are degrees of freedom.

### Correlation of log crowding distance across visual field, crowding orientation, and target kind

**Figure 10.**
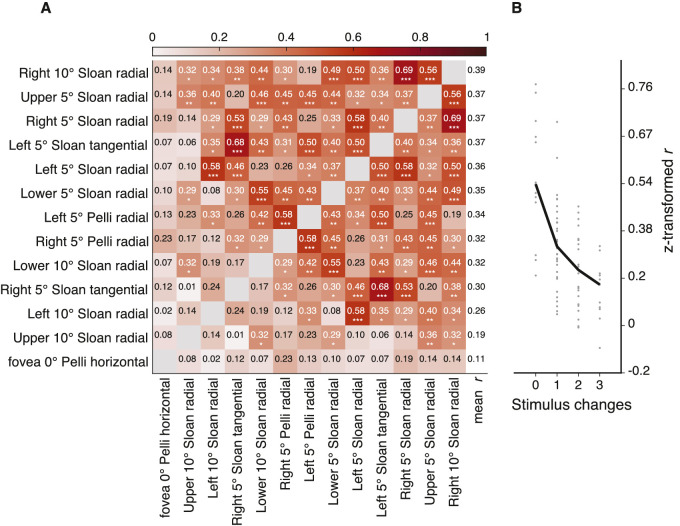
Correlations of log crowding distance. (**A**) Pair-wise correlations between crowding distances for various conditions (Table 1). Rows are sorted so that the average correlation decreases from top to bottom. (**B**) Average correlation as a function of the number of stimulus property differences in radial eccentricity, meridian, crowding orientation, and target kind. For example, comparing two eccentricities is a one-parameter difference.

We explored the pattern of correlations of log crowding distance for visual field locations, crowding orientations, and target kinds. These correlations are shown in Figure 10A, where each cell shows Pearson's *r* between two measurements. Rows are sorted so that the average correlation decreases from top to bottom. We found that log crowding distance measured on the right meridian at 10 deg eccentricity with the Sloan font and radial flankers yielded the highest average correlation with other log crowding distances (*r* = 0.39 with all, and *r* = 0.41 when fovea is excluded). Foveal log crowding distance measured with Pelli font yielded the smallest average correlation with the rest of the log crowding distances.

To summarize how correlation depends on stimulus properties we estimated the average correlation across measurements when one, two, or three stimulus properties (eccentricity, meridian, target kind, crowding orientation) differ (Figure 10B). The test–retest correlation of 0.54 is plotted at zero changes. The average correlation drops to 0.30 with one change, to 0.25 with two changes, and to 0.18 with three changes. We also estimated the average correlation at the same stimulus location, and we only varied font and crowding orientation (right or left meridian at 5 deg). We found an average correlation (across two changes) of *r* = 0.54. On the other hand, when we changed the location only and kept the stimulus properties (e.g., radial flankers, Sloan font, 5 deg) we obtained a much lower correlation of *r* = 0.32. This indicates that, when correlating crowding distances, location matters more than any other stimulus property.

### Correlation of Bouma factor *b* and intercept φ_0_

The Bouma law has 2 degrees of freedom, φ_0_ and *b*, which are anticorrelated, *r* = −0.51 (geometric mean across meridians).

### Standardized Bouma factor and its asymmetries across different studies

Visual field asymmetries can help identify the neural origin of perceptual phenomena (Afraz, Pashkam, & Cavanagh, 2010; Himmelberg, Winawer, & Carrasco, 2023). We compared our estimates of Bouma factor to all the previous studies that measured crowding asymmetry.

#### Estimating slope from just one point

The Bouma law has 2 degrees of freedom, the slope *b* and the negative intercept φ_0_. Estimating two parameters requires two measurements, but many crowding studies report crowding distance at only one eccentricity. In the complete case, we have thresholds *s*_0_ and *s* at eccentricities 0 and φ, and we use the definition of the Bouma factor *b* as the slope *b* = (*s* – *s*_0_)/(φ – 0). In the incomplete case, we have only threshold *s* at eccentricity φ. One might try to estimate the missing foveal threshold *s*_0_ or negative intercept φ_0_, but the simplest thing to do is to neglect φ_0_ (pretend it is zero), and estimate $\hat{b}=s/\varphi$. The estimate has fractional error $\in \;=\;\left(\hat{b}-b\right)/b=\varphi_{0}/\varphi$*.* Thus, neglecting φ_0_, possibly because the foveal threshold is unknown, leads to a fractional error φ_0_/φ. The studies in Table 2 used eccentricities φ ≥ 2 deg. Our measurements estimated φ_0_ = 0.24 deg. Thus, at 2 deg or beyond, the fractional error in the estimated Bouma factor will be at most 0.24/2 = 12%. The fractional error drops to 5% at 5 deg and to 2% at 10 deg.

**Figure 11.**
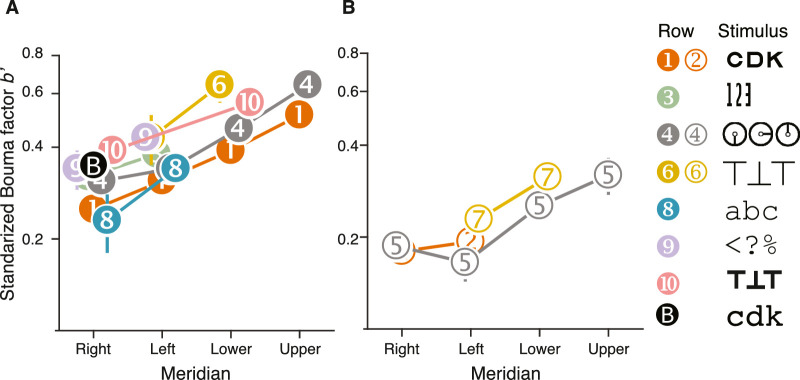
Standardized Bouma factor versus meridian for various studies and target kinds. Each numbered point corresponds to a numbered row of data in Table 6. (**A**) Comparison of radial-crowding studies and (**B**) tangential-crowding studies. Both panels plot standardized Bouma factor versus meridian. The legend shows the crowding stimuli. The white-on-black *B* symbol is the standardized Bouma factor estimated from Bouma (1970) data at 4 deg eccentricity with a 75% threshold criterion, with help from the reanalysis in Coates et al. (2021).

Each Bouma factor was multiplied by a correction factor to account for criterion differences and log versus linear flanker spacing (Table 2). Correction provides a standardized Bouma factor *b*′ for each study (Table 6). Figure 11 compares crowding across studies by plotting the standardized Bouma factor vs. meridian.

**Table 6.** Standardized Bouma factor versus meridian across studies. We compared our results with those of four studies that estimated crowding distance on at least two of the four cardinal meridians and reported the Bouma factor by dividing crowding distance by the eccentricity of the target. We standardized each Bouma factor by correcting for differences in the number of target choices and threshold criterion (see last column of Table 2). Data from Greenwood et al. (2017) and Coates et al. (2021) were obtained through personal communication. Data from Toet and Levi (1992) and Grainger et al. (2010) were digitized from figures in their papers. Data from Bouma (1970) were replotted from Coates et al. (2021). We estimated the standard error of the Bouma factors in the Grainger et al. (2010) study based on the *p* value they reported for meridian differences.

						Mean standardized Bouma factor (*b*′) (95% CI)			
Row	Study	*N*	Gaze tracking	Target	Crowding orientation	Right	Left	Lower	Upper
1	Our data	50	Yes	Sloan font	Radial	0.239 (0.22–0.25)	0.308 (0.30–0.32)	0.390 (0.38–0.40)	0.495 (0.48–0.49)
2	Our data	50	Yes	Sloan font	Tangential	0.143 (0.14–0.15)	0.154 (0.14–0.15)	—	—
3	Our data	50	Yes	Pelli font	Radial	0.325 (0.31–0.34)	0.377 (0.36–0.39)	—	—
4	Greenwood et al. (2017)	12	Yes	Tumbling clock	Radial	0.313 (0.28–0.33)	0.343 (0.30–0.38)	0.464 (0.42–0.51)	0.636 (0.59–0.70)
5	Greenwood et al. (2017)	12	Yes	Tumbling clock	Tangential	0.172 (0.16–0.19)	0.152 (0.13–0.17)	0.232 (0.20–0.25)	0.293 (0.26–0.33)
6	Toet and Levi (1992)	6	No	Tumbling T	Radial	—	0.426 (0.36–0.51)	0.638 (0.57–0.72)	—
7	Toet and Levi (1992)	6	No	Tumbling T	Tangential	—	0.213 (0.20–0.23)	0.293 (0.27–0.32)	—
8	Grainger et al. (2010)	27	No	Courier New letter	Radial	0.227 (0.19–0.28)	0.340 (0.27–0.37)	—	—
9	Grainger et al. (2010)	27	No	Courier New symbol	Radial	0.340 (0.29–0.39)	0.433 (0.38–0.48)	—	—
10	Coates et al. (2021)	4	No	Tumbling T	Radial	0.386 (0.37–0.40)	—	0.559 (0.55–0.57)	—
B	Bouma (1970)	25	No	Courier 10	Radial	0.35	—	—	—

#### Effects of meridian and target kind

Two rows in Table 6 (1, Sloan letter; 4, tumbling clock) report radial standardized Bouma factors for all four cardinal meridians. Figure 11A shows that, although the standardized Bouma factor was higher for the clock than for Sloan by a factor of 1.3 (ratio of means for the right meridian 0.313/0.239 = 1.3), the two curves are otherwise similar, showing the same dependence on meridian. The 1.3:1 difference is not an artifact of number of choices or threshold criterion (Table 2). Both studies used gaze tracking to exclude fixation errors, so the difference is not a consequence of bad fixation. Thus, this seems to be a real 1.3:1 difference in standardized Bouma factor between target kinds, precisely what the target-kind factor *t*_kind_ is meant to account for in Equation 13. The tumbling clocks may be more like each other than the nine Sloan letters are and therefore produce larger crowding distance. Almost all other studies (rows 3, 6, 9, and 10) cluster above the Sloan font and show the same dependence on meridian. In general, we found that Courier New letters (row 8) produced the smallest radial standardized Bouma factor (0.23 on the right meridian) and tumbling Ts (row 10) produced the largest radial standardized Bouma factor (0.39 on the right meridian).

#### Tangential crowding

We also compared standardized Bouma factors estimated with tangential flankers across studies (Figure 11B). The tangential Bouma factor did not vary as much as radial, especially in the left meridian (Figure 11B, rows 2, 5, and 7). Radial crowding estimates, even with the same stimuli, showed more variation in the standardized Bouma factor (Figure 11A, rows 1, 4, and 6). Although our data did not show any difference between right and left meridians (row 2), data extracted from Greenwood et al. (2017) do show a slight right:left advantage (row 5).

#### Meridional asymmetries

**Figure 12.**
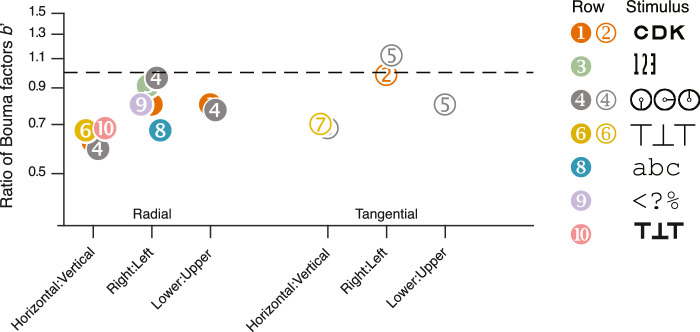
Plot of the three asymmetries, expressed as the ratio of Bouma factors. Each numbered point corresponds to a numbered row of data in Table 7. The horizontal dashed line at 1 represents no asymmetry. Each point is a ratio between Bouma factors. As in Figure 11, the legend shows examples of the objects that were used to measure crowding distance.

Table 7 and Figure 12 report three Bouma factor asymmetries (horizontal:vertical, right:left, and, lower:upper Bouma factor ratios) for our and four selected studies. On average, the Bouma factor asymmetry is larger radially than tangentially. Radially, there is an advantage of horizontal over vertical meridian, right over left meridian, and lower over upper meridian in every study. The horizontal:vertical advantage seems to be insensitive to object kind as the estimates are clustered around ratios of 0.6 to 0.7. Similarly, the lower visual field advantage is close to 0.8 for both studies that tested at this location (rows 1 and 4). The right:left asymmetry is the most variable. The right:left ratio is smallest for Courier New letters (row 8) and largest for clocks (row 4).

**Table 7.** The Bouma factor and its asymmetries, compared with the literature. As in Table 6, we compared our results with those of studies that measured crowding distance on more than one of the four cardinal meridians. Each row lists the target type, crowding orientation, and mean ratio with standard-error intervals across participants.

						Ratio of Bouma factors (95% CI)		
Row	Study	*N*	Gaze tracking	Target	Crowding orientation	Horizontal:vertical	Right:left	Lower:upper
1	Our data	50	Yes	Sloan font	Radial	0.62 (0.60–0.64)	0.78 (0.78–0.91)	0.79 (0.76–0.82)
2	Our data	50	Yes	Sloan font	Tangential	—	0.93 (0.93–0.96)	—
3	Our data	50	Yes	Pelli font	Radial	—	0.86 (0.82–0.90)	—
4	Greenwood et al. (2017)	12	Yes	Tumbling clock	Radial	0.59 (0.57–0.64)	0.91 (0.88–1.05)	0.73 (0.70–0.84)
5	Greenwood et al. (2017)	12	Yes	Tumbling clock	Tangential	0.68 (0.6–0.75)	1.13 (0.97–1.40)	0.79 (0.73–0.91)
6	Toet & Levi (1992)	6	—	Tumbling T	Radial	0.67 (0.61–0.78)	—	—
7	Toet & Levi (1992)	6	—	Tumbling T	Tangential	0.73 (0.67–0.84)	—	—
8	Grainger et al. (2010)	27	—	Courier New letter	Radial	—	0.67 (0.56–0.76)	—
9	Grainger et al. (2010)	27	—	Courier New symbol	Radial	—	0.79 (0.70–0.90)	—
10	Coates et al. (2021)	4	—	Tumbling T	Radial	0.69 (0.66–0.70)	—	—

### What does peripheral crowding distance add to foveal acuity?

#### Not predicted by acuity

**Figure 13.**
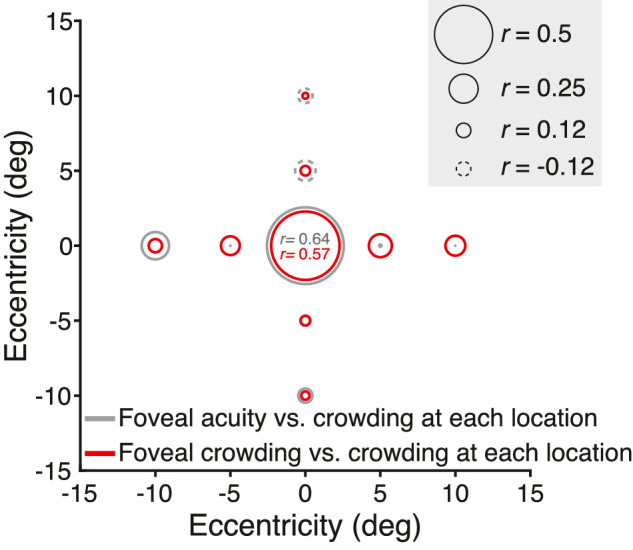
Foveal acuity and crowding fail to predict peripheral crowding. Correlation of foveal acuity (grey) and foveal crowding (red) with crowding everywhere. The central circles are test–retest for acuity (black) and crowding (red).

Any evaluation of the usefulness of crowding distance as a biomarker must assess what crowding tells us about the observer over and above what can be gleaned from foveal acuity, which is routinely measured in all optometric and ophthalmic exams. For our 50 observers, foveal acuity failed to predict peripheral crowding, with an insignificant average correlation of 0.04 (Figure 13, gray peripheral circles). More generally, both acuity and crowding measured in the fovea fail to predict peripheral crowding (average foveal–peripheral crowding correlation is an insignificant correlation of 0.15) (Figure 13, red circles). Within the fovea, we do find a significant correlation between acuity and crowding (*r* = 0.64). (It may be mere coincidence, since they are different measures made with different fonts, but we were struck by the near equality of geo. mean foveal size and spacing thresholds: 0.07 deg acuity and 0.08 deg crowding distance, Figure 8). Thus, foveal acuity predicts foveal crowding but not peripheral crowding. If peripheral crowding is of interest (e.g., as a possible limit to reading speed), then it should be measured, as it is not predicted by foveal acuity.

#### Foveal acuity and crowding

Our procedure measures threshold by covarying size and spacing. Because we found that foveal acuity and crowding are correlated, one might ask whether the correlation is due to the measured crowding threshold being contaminated by acuity limits. For five observers, using the same CriticalSpacing.m software, Pelli et al. (2016) measured foveal spacing threshold with the Pelli font with several spacing:size ratios and, for each observer, confirmed that all of the measured thresholds corresponded to one spacing at different sizes. For those five normally sighted observers, this showed that the procedure measured a crowding threshold, not acuity.

### The peeking-observer model

#### Without gaze tracker

We wondered how the Bouma factor estimate depends on fixation accuracy and we wondered if fixation accuracy might explain the difference between the two Bouma factor histograms in Figure 1. Peripheral identification is difficult, so, in ordinary life, we typically first foveate a peripheral target that we need to identify. Despite instructing observers to fixate on the central cross, we know that gaze could be elsewhere during the target presentation. The observer is torn between the desire to follow the instruction to fixate the cross and the natural impulse to fixate an anticipated peripheral target location. When the target location is randomly one of several peripheral locations, the participant's anticipation of location is often wrong. We modeled the participant's gaze position by two distributions, one without and one with peeking. First, for the no-peeking awaited-fixation distribution, we used the measured eye position in the awaited-fixation dataset, in which the participant's gaze was within 1.5 deg of the crosshair center for 250 ms immediately before target presentation, and we discarded trials in which gaze was more than 1.5 deg from the crosshair center during target presentation. The awaited-fixation gaze-position distribution was compact and roughly centered on the fixation crosshair. Second, we considered peeking toward a possible target location. We supposed that the participant peeks on a fraction *p* of the trials, and that the peeking eye movement travels only a fraction *k* of the distance from the crosshair to the possible target location, with a Gaussian error (0.5 deg *SD* in *x* and in *y*). The peeking distribution has a mode corresponding to each possible target location, but at a fraction *k* of the possible target eccentricity. Gaze position is randomly sampled from the peeking distribution on a proportion *p* of trials and otherwise from the awaited-fixation distribution.

In the spirit of the Bouma law, our peeking-observer model assumes that the probability of identifying the target is given by a psychometric function:

(16) $$\begin{array}{c} P\left(r\right)=1-0.5\;\exp\left(-{\left(\frac{r}{b_{\mathrm{true}}}\right)}^{\beta }\right)\; \end{array}$$

that depends solely on the ratio *r* of target-flanker spacing to actual target eccentricity, where *b*_true_ is the true Bouma factor and steepness β is 2.3. For simplicity, the model omits threshold criterion and finger-error probability delta. Bouma factor *b* is estimated by 35 trials of QUEST, assuming the true psychometric function, with a prior guess = 0.11 of *r* and an assumed *SD* = 2 of log *r.*

#### Awaited*-*fixation distribution

The awaited*-*fixation distribution was 3500 actual gaze positions at stimulus onset (35 trials × 50 participants × 2 sessions) measured with an EyeLink eye tracker in our awaited*-*fixation dataset. Recall that the stimulus was presented only when the gaze had been within 1.5 deg of the crosshair center for 250 ms.

#### Peeking distribution

**Figure 14.**
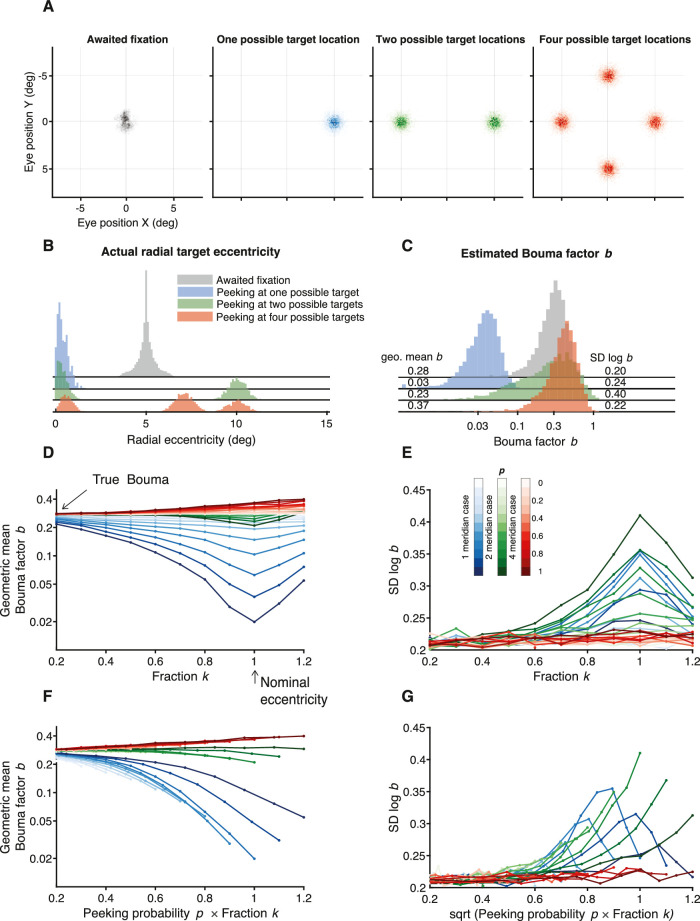
The peeking model. (**A**) Scatter diagrams of the distribution of gaze position for each number of possible target locations. The gray histogram was measured by the EyeLink eye tracker in the awaited-fixation dataset. The green, blue, and red distributions of gaze position were synthesized assuming a full peek fraction *k* = 1 and a Gaussian (*SD* = 0.5 in *x* and 0.5 deg in *y*) centered at each possible target location (**B**) Four histograms showing “actual” radial eccentricity of the target based on the distributions in (**A**). “Actual” indicates that target eccentricity is computed relative to gaze position rather than the crosshair that the observer was asked to fixate. (**C**) Five thousand Bouma factors estimated using QUEST (35 trials) where each trial used the actual eccentricity. (**D**) Geometric mean of the Bouma factor for each simulated case plotted versus fraction *k*. The arrow on the vertical axis indicates the true value of the Bouma factor that we assumed (0.3), and the arrow on the horizontal axis shows nominal eccentricity (5 deg). (**E**) *SD* of Bouma factor versus fraction *k*. Color saturation indicates the percentage of trials in which observers’ peeked (see inset color bar). (**F**, **G**) Same as (**D**) and (**E**); however, the geometric mean of the Bouma factor and its *SD* are plotted versus the combination of peeking probability *p* with fraction *k*.

We considered one, two, and four possible target locations (Figure 14A). First, the target was always presented at one location (right meridian at 5 deg). Second, the target was randomly presented at ±5 deg on the horizontal midline. Third, the target was at 5 deg radial eccentricity on a random one of the four cardinal meridians (right, left, upper, lower). When participants “peek” at a possible target location, depending on the number of possible locations, they have a 100%, 50%, or 25% chance of selecting the target location. Target and gaze position together define the actual target eccentricity.

Nominal eccentricity of the target is relative to the crosshair. Actual eccentricity of the target is relative to gaze position when the target is presented. Peeking near the target position will reduce the actual eccentricity to practically zero, whereas peeking another target location could result in an actual eccentricity greater than nominal. Figure 14B shows the actual target eccentricities calculated based on the four distributions of gaze position in Figure 14A.

The nominal radial eccentricity of the target is always 5 deg, so we simulated an observer with a threshold spacing of 1.5 deg by using the Weibull psychometric function that is assumed by QUEST during threshold estimation (with β = 2.30, δ = 0.01, γ = 0.11). Thus, our model assumes a true Bouma factor of 1.5/5 = 0.3. The point of this exercise is to evaluate how various methods estimate Bouma factor. We simulated a block of 35 trials using QUEST to estimate the 70% correct spacing threshold from which we could calculate Bouma factor. We repeated this many times to obtain a histogram of estimated Bouma factors (Figure 14C) for awaited fixation (gray) and peeking (colored) distributions.

The modeling shows that the geometric mean estimated Bouma factor *b* was lowest (0.03) for peeking with one possible location and highest with four (0.37), given a true Bouma factor *b *= 0.30. With no peeking (*p* = 0), gaze position was from the awaited*-*fixation distribution, and the model estimate of Bouma factor was 0.28, very close to the true value of 0.30. The *SD* of log Bouma factor *b* was highest (0.40) with two possible locations and lowest (0.22) with four.

The error (deviation from the assumed Bouma factor *b* = 0.3; see arrow on the vertical axis in Figure 14D) in estimating Bouma factor grows with the proportion *p* and fraction *k* of peeking (Figure 14D). Observing that the error of the estimated Bouma factor grows proportionally with *k* and that its *SD* grows proportionally with *k*^2^ (note the parabolic shape in Figure 14E), we produced new plots (Figures 14F, 14G) showing that the geometric mean of *b* is roughly linear with *p* × *k* and the *SD* of log *b* is roughly linear with $\sqrt{p\;\times \;k}$.

**Figure 15.**
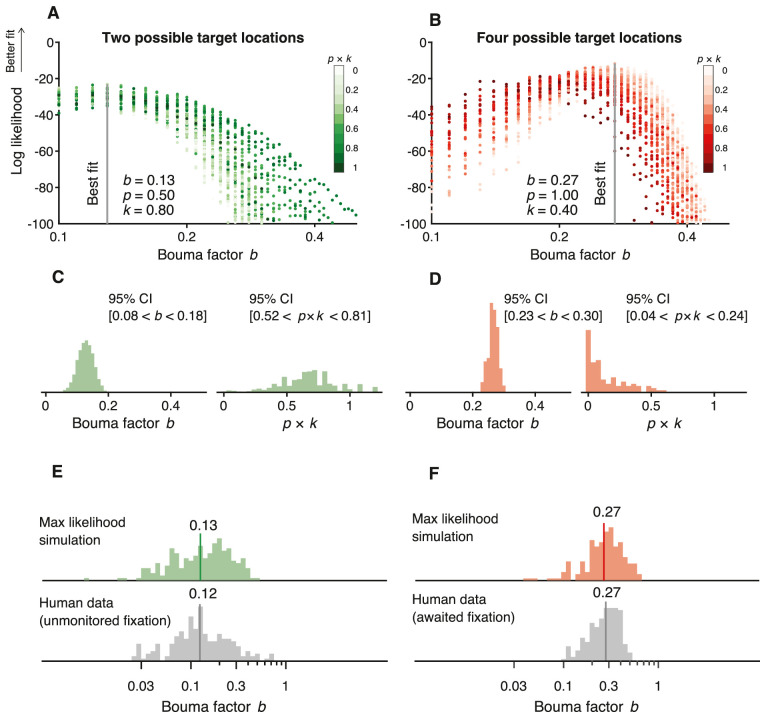
Comparing the peeking model with data. We used log-likelihood estimation to find the model parameter values that best fit our data, where the higher the likelihood, the better the fit. (**A**, **B**) Log-likelihood is plotted versus estimated Bouma factor *b*. A vertical gray line indicates the best fit. Plots elsewhere use the best-fitting value. Brightness indicates the product *p × k*. (**C**, **D**) Bootstrapped model estimates. For each model we bootstrapped the fit (*n* = 100) by randomly removing 25% of the data at each iteration and fitting the model on the remaining data. Each histogram contains 100 best-fitted parameters from each iteration. The *p* × *k* confidence intervals were calculated after bootstrapping the *p* × *k* parameter (*n* = 1000) and averaging 10 random samples at each iteration. This created a normal distribution and allowed for the calculation of confidence intervals. (E, F) Comparison of the histograms of best-fitting simulated and acquired data. The solid vertical line indicates the geometric mean. For unmonitored fixation, the geometric mean of Bouma factor *b* was 0.13 for the simulated data and 0.12 for the human data. The *SD* of log Bouma factor was 0.32 for the simulated data and 0.31 for the human data. For awaited fixation, the geometric mean of Bouma factor was 0.27 for the simulated data and 0.27 for the human data. The *SD* of log Bouma factor was 0.21 for the simulated data and 0.19 for the human data. Note that the red distribution in (D) is different from the red distribution in Figure 1. Here, we plotted data from all four meridians. Data plotted in Figure 1 are extracted from just the right and left meridians.

Given *p*, *k*, and the true Bouma factor, our peeking-observer model predicts the estimated Bouma factor *b*. We estimated *p* and *k* by fitting the model using maximum likelihood optimization. The higher the log-likelihood, the better the fit. We wanted to determine how often (*p*) and how far (*k*) the observer peeks and the estimated Bouma factor *b.* In Figures 15A and 15B, the scatter and breadth of the log-likelihood distribution result in a broad confidence interval for the product *p* × *k*. To estimate the error of the fit, we bootstrapped it by removing 25% of data at each iteration (*n* = 100). For unmonitored fixation (Figure 15A), the bootstrapped parameters were consistent with high peeking (0.5 < *p* × *k* ≤ 1) and rejected no peeking (*p* × *k* = 0). For awaited fixation (Figure 15B), the bootstrapped parameters were consistent with low peeking (*p* × *k <* 0.5) and rejected high peeking (*p* × *k* = 1). For each dataset, Figures 15E and 15F show that the human data are well matched by the simulated histogram. The two geometric means of *b* match, as do the standard deviations of log *b*. Our peeking model of eye position and crowding predicts the estimated Bouma factor in both cases, showing that the unmonitored fixation results are well fit by high peeking, and the awaited fixation results are well fit by low peeking. One simple model of eye position and crowding fits all our data.

It is our impression that as observers gain experience with peripheral viewing, they peek less. Based on Figure 15, Bouma's (1970) peeking rate must have been practically zero.

## Discussion

Levi (2008), Pelli and Tillman (2008), Herzog et al. (2015), Strasburger (2020), and Coates et al. (2021) have reviewed the crowding literature. Most recently, Coates et al. (2021) provided a compact summary of the effects on the Bouma factor of contrast, size, target–flanker similarity and visual field location. This summary includes reanalysis of old data and shows a weak effect of stimulus duration. They also measured new data with two durations and two meridians confirming the effect of duration on the Bouma factor. Most of the data in their paper were acquired on fewer than five observers. We measured the effect of meridian, eccentricity, crowding orientation, and font with 50 observers. We did not measure effects of contrast, duration, or target-flanker similarity, but otherwise we confirm all the effects that they reported. We provide an equation predicting how crowding distance depends on meridian, target kind, and crowding orientation for each observer. We also show that crowding is reliable across days.

### Bouma law and factor

#### Bouma law

The Bouma law describes the linear increase of crowding distance with eccentricity (Bouma, 1970; Bouma, 1973; Levi, 2008; Pelli & Tillman, 2008; Rosen et al., 2014). Bouma factor is the slope of that line (Rosen et al., 2014). Bouma law is robust when fit to individual observer's data (Pelli et al., 2004; Rosen et al., 2014; Strasburger, 2020; Strasburger et al., 1991). In this study, for the first time, we fit Bouma law to data that include measurements from 50 observers tested with two crowding orientations at nine locations of the visual field. Bouma law is an excellent fit to our data and explains 82.5% of the variance despite being just a straight line with 2 degrees of freedom. We tried adding terms to Bouma law to account for known factors: crowding orientation (Greenwood et al., 2017; Kwon et al., 2014; Petrov & Meleshkevich, 2011; Toet & Levi, 1992), meridional location of the stimulus (Fortenbaugh et al., 2015; Greenwood et al., 2017; He et al., 1996), target kind (Coates et al., 2021; Grainger et al., 2010), and individual differences (Petrov & Meleshkevich, 2011; Veríssimo, Hölsken, & Olivers, 2021). We found that the enhanced model explains a bit more variance (increased from 82.5% to 94%). Eccentricity remains the dominant factor, accounting for 82.5% of the variance.

#### Standardized Bouma factor

We define the standardized Bouma factor *b*′ as the reported Bouma factor *b* (ratio of crowding distance to radial eccentricity) multiplied by a correction factor that account for differences in task from Bouma's 25 choice alternatives, 75% threshold criterion, and linear flanker symmetry. Bouma reported a “roughly” 0.5 slope for radial letter crowding versus eccentricity (Bouma, 1970). Andriessen and Bouma (1976) later reported a slope of 0.4 for crowding of lines. Coates et al. (2021) reanalyzed Bouma's original data with various threshold criteria so we interpolated between the 70% and 80% thresholds to estimate the 75% threshold. Estimating Bouma factor from Bouma's original data using this criterion yielded a Bouma factor of 0.35, in line with modern estimates of 0.3 (Table 6 and Supplementary Table S2). Figure 11A shows that the corrected Bouma factor *b*′ ranges from 0.23 for Courier New letters to 0.39 for tumbling T measured with radial flankers on the right meridian. That residual difference may be due to target kind (Coates et al., 2021; Grainger et al., 2010). This is further supported by our finding that Bouma factor was 0.78 lower for the Sloan font than for the Pelli font.

### Supralinearity and the Bouma law

The linearity of the Bouma law implies that the Bouma factor is independent of eccentricity. The Coates et al. (2021) reanalysis of Bouma's 1970 data found a twofold increase of the Bouma factor with eccentricity (from 1 to 7 deg), with a log-log slope of 0.35. Coates et al. (2021) speculated that this eccentricity dependence might be due to Bouma's use of constant size stimuli at all eccentricities. Both acuity and crowding can limit measured thresholds for size and spacing across the visual field (Pelli et al., 2016; Song et al., 2014). If the threshold is independent of size, it is a crowding threshold; if the threshold is independent of spacing, it is an acuity threshold. In 10 participants, we measured crowding distance at eccentricities of 0, 5, 10, 20, and 30 deg, scaling letter size with spacing as is now usual (see Methods). In our results with proportional letter size and controlled eye position, we found a similar twofold increase of the Bouma factor with eccentricity (from 5 to 30 deg), with a log-log slope of 0.38. Enhancing the Bouma law to allow a nonlinear dependence on eccentricity improves the fit to 10 observers’ data slightly, increasing the variance accounted for from 90% to 95%. This effect is small but detectable in data from 0, 5, and 10 deg (Figure 9A) and becomes pronounced at eccentricities of 20 and 30 deg (Figure 9B). To our knowledge, only a few past studies measured crowding beyond 10 deg eccentricity (Bouma, 1970; Kalpadakis-Smith, Tailor, Dahlmann-Noor, & Greenwood, 2022; Kwon & Liu, 2019; Pelli et al., 2004), and all these datasets show supralinear growth with eccentricity. From the perspective of mathematical modeling, Bouma initially suggested a simple proportionality with one term, which later was extended to linearity with two terms, and the evidence for supralinearity justifies a three-term quadratic polynomial. Biologically, it seems possible that the increase of the Bouma factor at high eccentricity reflects a compression of eccentric visual field in higher order areas. Indeed, hV4 has a reduced peripheral representation when compared with earlier visual areas, V1, V2, and V3 (Arcaro, McMains, Singer, & Kastner, 2009; Goddard, Mannion, McDonald, Solomon, & Clifford, 2011; Kolster, Peeters, & Orban, 2010; Winawer & Witthoft, 2015). This parallels the idea that the ventral visual stream, specialized in object recognition, emphasizes the central visual field (Levy, Hasson, Avidan, Hendler, & Malach, 2001; Ungerleider & Haxby, 1994).

### Crowding asymmetries

At any given eccentricity, the Bouma factor varies with polar angle. The Bouma factor is lower along the horizontal than vertical meridian (Greenwood et al., 2017; Petrov & Meleshkevich, 2011; Toet & Levi, 1992), is higher in the upper meridian than the lower meridian (Fortenbaugh et al., 2015; Greenwood et al., 2017; He et al., 1996; Toet & Levi, 1992), tends to be lower in the right meridian than the left meridian (Grainger et al., 2010; White, Tang, & Yeatman, 2020), and approximately halves with tangential flankers (Greenwood et al., 2017; Kwon et al., 2014). In this work, we replicated all of these asymmetries (Figure 12 and Table 7). The horizontal versus vertical advantage and better performance in the lower versus upper visual field is found for many visual tasks (Himmelberg et al., 2023), and these asymmetries parallel those found in population receptive field size, cortical magnification, retinal ganglion cell density, and the BOLD signal magnitude (Benson, Kupers, Barbot, Carrasco, & Winawer, 2021; Himmelberg, et al., 2021; Kupers, Benson, Carrasco, & Winawer, 2022; Kupers, Carrasco, & Winawer, 2019; Kurzawski, Gulban, Jamison, Winawer, & Kay, 2022; Kwon & Liu, 2019; Liu, Heeger, & Carrasco, 2006; Silva, et al., 2018). The right:left asymmetry seems to be least described and does not generalize across all tasks. Beyond crowding, right visual field advantages have been reported: For native readers of left-to-right written languages, such as English, the right meridian outperforms left in word recognition (Mishkin & Gorgays, 1952). Worrall and Coles (1976) examined letter recognition across the visual field and found a significant right hemifield advantage only along the right horizontal midline. The similarities in asymmetry suggest a common mechanism, and the differences may be useful hints toward the cortical substrate of crowding.

### Standard deviation of measured acuity and crowding

To estimate the reliability of our measurements, we acquired each threshold twice. Previous work showed improved performance in crowding tasks for repeated measurements (Chung, 2007; Malania, Pawellek, Plank, & Greenlee, 2020). From their figures, we estimated the second-block benefit to be 13% for Malania et al. (2020) and 20% for Chung (2007). (Chung showed thresholds before and after 60- 100-trial blocks and showed percent correct for each block. By eye, we estimate that the benefit from first to second block is about a third of that provided by the 60 blocks of training; thus, her 62% advantage [see average data from Table 1 in Chung, 2007] after 60 blocks corresponds to the 20% advantage after the first block.) We found a modest second-threshold improvement for crowding thresholds measured with the Sloan font at all tested locations and with the Pelli font in the fovea (less than 10%). Thresholds measured with the Pelli font in the periphery yielded the highest improvement (23%). In our data, the improvement is likely not due to acquiring familiarity with the task, as all observers participated in a training session, which consisted of repeated trials until 10 answers are correct. We found no improvement in acuity.

Overall, we found very good reproducibility of crowding and acuity thresholds (Figure 5). The standard deviation of log Bouma factor *b* measured with the Sloan font and radial flankers for test–retest is much lower than the standard deviation of log Bouma factor across observers (0.03 vs. 0.08).

### Individual differences

Estimating individual differences requires data from many observers. In this paper, we measured crowding in 50 observers, which is the biggest dataset of crowding measurements to date. Previous in-person crowding surveys included at most 27 observers (Grainger et al., 2010; Greenwood et al., 2017; Petrov & Meleshkevich, 2011; Toet & Levi, 1992). (An online crowding study tested 793 observers, but did not report individual differences; see Liu et al., 2009.) To capture individual differences in Bouma factor, we included an observer factor, *o_i_*, in the enhanced model. Adding the observer factor improved the explained variance from 92.6% to 94% (Table 4). Although this effect may seem negligible at first, we found that the Bouma factor varied twofold across observers, ranging from 0.20 to 0.38 (Figure 8A). A similar, twofold variation was observed for all other thresholds that we estimated (Figure 8A).

### The Bouma factor as a biomarker

Large individual differences enhance the potential for crowding to serve as a biomarker for studying cortical health and development. Specifically, crowding varies across children, too (Kalpadakis-Smith et al., 2022), and predicts rapid serial visual presentation (RSVP) reading speed (Pelli et al., 2007). Foveal crowding distance drops threefold from age 3 to 8 years (Waugh et al., 2018). If crowding correlates with the reading speed of beginning readers, then preliterate measures of crowding might help identify the children who need extra help before they learn to read. Measuring crowding distance across individuals in several diverse populations might expose any limit that crowding imposes on reading, yielding a norm for the development of crowding. Huge public interventions seek to help dyslexic children read faster and to identify them sooner. A virtue of crowding distance as a potential biomarker for dyslexia and cortical health is that it can be precisely measured in 30 minutes.

### Crowding correlations

This paper reports 13 crowding thresholds for each of 50 observers. Such a comprehensive dataset allows for a correlation analysis to assess how well each crowding threshold predicts the others. We found a moderate correlation of crowding between peripheral locations (*r* = 0.39 averaged across all peripheral locations) and hardly any between fovea and periphery (*r* = 0.11). We also found that crowding measured with radial flankers correlated highly with crowding measured with tangential flankers at the same location (*r* = 0.53 for the right meridian, *r* = 0.50 for the left meridian). The threshold measurement that best predicted all other peripheral thresholds (excluding the fovea), with a correlation *r* = 0.41, was radial Sloan crowding at 10 deg in the right meridian.

#### Effect of stimulus configuration vs. location

We found higher correlation (*r* = 0.54) when the location was the same and the stimulus configuration was changed, than (*r* = 0.32) when the stimulus configuration was the same and the location changed. Correlation of crowding distance depends more on location than configuration. Paralleling our result, Poggel and Strasburger (2004) found only a weak correlation across meridians for visual reaction times. Surprisingly little is known about the spatial correlations of basic measures such acuity and contrast sensitivity.

### The peeking-observer model

We always asked the observer to fixate on the crosshair during each trial. We acquired data with two methods: unmonitored fixation, without gaze tracking, and awaited fixation, in which the stimulus was only presented when gaze was near the fixation cross. Both methods are described in detail in the Methods section. The two methods yielded different Bouma factor distributions. Upgrading from unmonitored to awaited fixation increased the Bouma factor mean *b* from 0.12 to 0.20 and nearly halved the *SD* of log *b* from 0.31 to 0.18. Histograms are shown in Figure 1. This peeking-observer model assumes, first, that performance on each trial depends solely on target eccentricity (relative to gaze position); second, that the observer peeks on a fraction *p* of the trials and fixates near the crosshair on the rest of the trials; and, third, that the location of the peek is a fraction *k* of the distance from the fixation mark to the anticipated target location.

In unmonitored fixation, the observer peeks with probability *p*. In awaited fixation, peeking is prevented by using gaze-contingent display and discarding any trials where gaze left the fixation cross while the target was present. Suppose there are two possible target locations. The Bouma factor distribution is unimodal for low values of *p* and becomes bimodal for high values for *p*. Our unmonitored *b* histogram is bimodal and is best fit with a peeking probability of 50%. Our awaited*-*fixation *b* histogram is unimodal and best fit by peeking restricted to the 1.5 deg from the crosshair allowed by the gaze tracker. Upgrading from the bimodal to the unimodal *b* distribution raised the mean *b* from 0.12 to 0.27 and nearly halved the standard deviation of log *b* from 0.31 to 0.19.

The peeking model does not account for the reduction of the crowding distance of a target that occurs in anticipation of a saccade to the target (Harrison, Mattingley, & Remington, 2013). It is conceivable that on some awaited-fixation trials the observer was planning an eye movement to the correct target location and that this reduced crowding before the eye moved.

#### Effects of duration and peeking

Coates et al. (2021) reanalyzed crowding data from 16 studies and presented a scatter diagram of Bouma factor versus stimulus duration. The plot of Bouma factor versus log stimulus duration had a semi-log slope of −0.16 describing how the Bouma factor drops with duration. Their analysis included many studies, with various threshold criteria, from various meridians, which introduced differences in the Bouma factor. To avoid these confounds, Coates et al. (2021) collected new data using a consistent threshold criterion and consistent locations. In their new results, increasing the duration from 67 to 500 ms decreased the Bouma factor by a factor of 1/1.6. However, none of these studies monitored fixation. Our Figure 1 shows that, relative to controlled fixation, peeking can reduce the Bouma factor by a factor of 1.6, which is the size of the decrease with duration reported by Coates et al. (2021). If the probability of peeking grows with duration, then peeking might explain their drop in Bouma factor with duration.

#### Preventing peeking

Awaited fixation eliminates peeking, as shown above, but it requires an eye tracker. Kurzawski et al. (2023) show that manual cursor tracking of a moving crosshair nearly abolishes peeking and can be used online without an eye tracker.

### Why measure crowding?

#### Peripheral crowding provides additional information about visual health

Acuity is the threshold size of a target for recognition, whereas crowding is a spacing threshold. Clinical assessment routinely includes foveal acuity and not crowding. Both limit recognition of everyday objects. Our results show that peripheral crowding is independent of foveal acuity and might be a useful biomarker of visual health. Specifically, peripheral crowding might predict dyslexia (Bouma & Legein, 1977; Martelli, Di Filippo, Spinelli, & Zoccolotti, 2009; O'Brien, Mansfield, & Legge, 2005). There are hints that crowding tends to be worse in dyslexia (Pelli et al., 2007). If crowding correlates with reading speed of beginning readers, then preliterate measures of peripheral crowding might help identify the children who need extra help before they learn to read.

#### What about foveal crowding?

In healthy individuals, foveal crowding correlates with foveal acuity, but there are some conditions in which the two are dissociated. Strabismic amblyopia makes crowding worse in the fovea, but not in the periphery (Song et al., 2014). This suggests that the fovea might be the most sensitive place to detect the increase in crowding associated with amblyopia. Traditional tests for crowding are mostly peripheral and use a fixation mark and a brief peripheral target, which are poorly suited for testing children and dementia patients whose attention may wander. Such participants will fixate much more reliably on a foveal target. We hope there will be clinical studies to assess the diagnostic benefit of measuring crowding.

## Conclusions

1. The well-known Bouma law—crowding distance depends linearly on radial eccentricity—explains 82% of the variance of log crowding distance, cross-validated. Our enhanced Bouma law, with factors for observer, meridian, and target kind, explains 94% of the variance, cross-validated. The very good fit states the central accomplishment of the paper and shows how well the linear Bouma law fits human data.
2. The Bouma factor varies twofold across observers, meridians, and crowding orientations.
3. Consistent with past reports, five asymmetries each confer an advantage expressed as a ratio of Bouma factors: 0.62 horizontal:vertical, 0.79 lower:upper, 0.78 right:left, 0.55 tangential:radial, and 0.78 Sloan font:Pelli font.
4. As noted above, the Bouma factor varies twofold across observers. Differences across observers are much larger than those of test–retest. The 0.08 *SD* of log Bouma factor across observers is nearly triple the 0.03 *SD* of test–retest when log *b* is measured in half an hour (2 eccentricities × 4 meridians = 8 thresholds and 8 × 35 deg = 280 trials).
5. The growth of crowding distance with eccentricity is supralinear, which becomes obvious when measurements extend out to 30 deg eccentricity. The linear fit is adequate for most purposes.
6. Crowding distance measured at 10 deg eccentricity along the right meridian is the best predictor of average crowding distance elsewhere (average *r* = 0.39).
7. Peripheral crowding is independent of foveal crowding and foveal acuity.
8. Simulations and data show that peeking can skew estimates of crowding (e.g., greatly decrease the mean or double the SD of log *b*). Thus it is important to minimize peeking, e.g. by using awaited fixation (with gaze tracking) or manual tracking of a moving crosshair (without gaze tracking).

## Supplementary Material

Supplement 1
