# Supplementary material for: The Bouma law accounts for crowding in 50 observers
Source: J Vis. 2023 Aug 4;23(8):6. doi: 10.1167/jov.23.8.6 (PMC10408772; doi:10.1167/jov.23.8.6)
Supplement: Supplement 1 [file jovi-23-8-6_s001.pdf]

Effect of equating either the linear or log spacing of two radial flankers

When measuring radial crowding, the target lies between two flankers on a radial line from fixation. Bouma spaced the flankers equally, and most investigators have followed suit. However, we spaced the flankers symmetrically on a logarithmic rather than linear scale. This raises the question of how to compare crowding distances between experiments that spaced the flankers linearly vs logarithmically. Given the Bouma law (**Eq. 10**) and assuming that crowding distance depends primarily on the flanker-to-flanker distance, and negligibly on the target position between them, we show here that the crowding distance is expected to be 1.18 times larger when measured with linearly-spaced flankers than with log-spaced flankers.

Specifically, for a target at φ we choose the two flanker eccentricities φ_{in} and φ_{out} so that φ is located between φ_{in} and φ_{out}.

$$\log(\varphi_0+\varphi) = [\log(\varphi_0+\varphi_{in}) + \log(\varphi_0+\varphi_{out})] / 2, \qquad \text{(S1)}$$

where φ_0=0.15 deg, and we report the inner spacing $s = \varphi-\varphi_{in}$. We can rearrange

$$\log(\varphi_0+\varphi_{out}) = 2 \log(\varphi_0+\varphi) - \log(\varphi_0+\varphi_{in}) \qquad \text{(S2)}$$

Thus, the two flankers are at different (linear) distances from the target, but we suppose that they are equally effective in crowding the target. We report the center-to-center spacing from the inner flanker to the target as the “spacing” s.

We now estimate the relation of crowding distances measured in these two ways. We suppose that degree of crowding is determined by the separation between flankers on the log scale, as expected from the Bouma law:

$$\Delta\Phi = \log(\varphi_0+\varphi_{out}) - \log(\varphi_0+\varphi_{in}) \qquad \text{(S3)}$$

If we are in the periphery, i.e. $\varphi_{in} > 1$, then φ_0=0.15 is negligible, and we can simplify,

$$\Delta\Phi \approx \log(\varphi_{out} / \varphi_{in}) \qquad \text{(S4)}$$

Of course, increasing spacing alleviates crowding, so the degree of crowding will drop as log spacing $\Delta\Phi$ grows. Note that this model is at best an approximation, as it neglects position of the target. We are using it solely to compare crowding for two different ways of centering the target between flankers so the two target positions won’t differ by much.

Linear flanker spacing: With flankers spaced symmetrically about the target on a linear scale, both at distance s from the target:

$$\Delta\Phi \approx \log (\varphi+s)/(\varphi-s) \qquad \text{(S5)}$$

Log flanker spacing: With flankers spaced symmetrically on a log scale, the log flanker-to-flanker spacing is twice the log target-to-flanker spacing:

$$\Delta\Phi \approx 2 \log \varphi/(\varphi\text{-s'}) \quad \text{(S6)}$$

Now we equate the two log flanker spacings, one with linearly symmetric spacing *s*, the other with log-symmetric spacing *s'*.

$$\log (\varphi+\text{s})/(\varphi\text{-s}) = 2 \log \varphi/(\varphi\text{-s'}) \quad \text{(S7)}$$

Solve for *s'*,

$$s' = \varphi - \varphi\ [(\varphi\text{-s})/(\varphi+\text{s})]^{0.5} \quad \text{(S8)}$$

Now substitute $b=s/\varphi$ and $b'=s'/\varphi$,

$$b' = 1 - [(1-b)/(1+b)]^{0.5} \quad \text{(S9)}$$

Figure S1 shows that, to a good approximation, this is a proportionality, with error of at most 0.014 over the relevant range $0 \le b \le 0.9$,

$$b' \approx 0.845\ b \quad \text{(S10)}$$

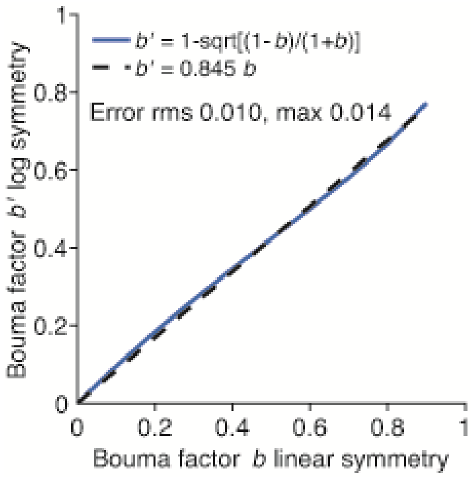

Figure S1. **Correspondence between linear and log flanker spacing.** Log-symmetric spacing $s'=\varphi b'$ for same crowding effect as each linearly symmetric spacing $s = \varphi b$.

Thus, our log-symmetric spacing $s'=\varphi b'$ is approximately 0.845 times the linearly-symmetric spacing $s=\varphi b$ that is traditionally reported.

Log-log versions of the Bouma models

The negative intercept φ_0 is small and negligible at large eccentricity. (Zeroing it in the Bouma law produces less than 5% error in predicted crowding distance at eccentricities beyond 4.8 deg.) If we consider only peripheral results (>4.8 deg eccentric) we can set $\varphi_0=0$, and express the Bouma models in log coordinates (Table S2). The multiplicative combination rule becomes additive in the new coordinates. As with the linear models, the cross-validated variance explained R^2 increases with more parameters. At large eccentricity, these models are equivalent to the linear models presented in the main text, but fitting is quicker because the fitting error can be minimized by linear regression.

Model	Equation	R^2 (%)	Pearson's R	No. of parameters
**Bouma law**	$\hat{S} \approx \Phi + B$	53.53	0.78	1
**× meridional factor**	$\hat{S} \approx \Phi + B_\theta$	71.38	0.85	4
**× crowding orientation**	$\hat{S} \approx \Phi + B_\theta + F_d$	80.47	0.89	6
**× target-kind factor**	$\hat{S} \approx \Phi + B_\theta + F_d + T_{kind}$	81.13	0.90	8
**× observer factor**	$\hat{S} \approx \Phi + B_\theta + F_d + T_{kind} + O_i$	84.32	0.92	58

Table S1 – Fitting the log-log version of the Bouma law (setting $\varphi_0=0$ and modeling only peripheral data $\varphi>4.8$ deg). Uppercase variables are the log10 of corresponding lowercase variables. R^2 represents variance explained after model cross-validation. All our fitting minimizes error in log crowding distance so fitting the log-log version can be fit using linear regression.

Correcting the Bouma factor

Comparison of crowding measured with different threshold criterion, number of choices, and log vs. linear spacing of flankers is facilitated by calculating the Standardized Bouma factor *b'*, which corrects for these factors. Each row number in Table S2 corresponds to a row in Table 6. The correction factors come from Table 2.

Row	Correction factor	Bouma factor<br>**Standardized Bouma factor**			
		**Right**	**Left**	**Lowe r**	**Upper**
1	**× 1.30 (1.10×1.18)**	0.184<br>**0.239**	0.237<br>**0.308**	0.300<br>**0.390**	0.381<br>**0.495**
3	**× 1.30**	0.25	0.29		

	**(1.10×1.18)**	**0.325**	**0.377**		
4	**× 1.01**	0.31 **0.313**	0.34 **0.343**	0.46 **0.464**	0.63 **0.636**
6	**× 1.33**		0.32 **0.426**	0.48 **0.638**	
8	**× 1.03**	0.22 **0.227**	0.33 **0.340**		
9	**× 1.03**	0.33 **0.340**	0.42 **0.433**		
10	**× 1.33**	0.29 **0.386**		0.42 **0.559**	
Geometric mean		0.26 **0.30**	0.32 **0.37**	0.40 **0.50**	0.49 **0.56**

Table S2 – Radial Bouma factor before and after the correction. Row numbers correspond to numbers in Table 6. Each cell contains two numbers. The upper number is the Bouma factor before accounting for measurement differences and the lower one is the Standardized Bouma factor (already multiplied by the correction factor). Correction factors are calculated in Table 2. The Standardized Bouma factor is overall higher (e.g., 0.26 to 0.32 on the right meridian).
